# Novel Murine Dendritic Cell Lines: A Powerful Auxiliary Tool for Dendritic Cell Research

**DOI:** 10.3389/fimmu.2012.00331

**Published:** 2012-11-07

**Authors:** Silvia A. Fuertes Marraco, Frédéric Grosjean, Anaïs Duval, Muriel Rosa, Christine Lavanchy, Devika Ashok, Sergio Haller, Luc A. Otten, Quynh-Giao Steiner, Patrick Descombes, Christian A. Luber, Felix Meissner, Matthias Mann, Lajos Szeles, Walter Reith, Hans Acha-Orbea

**Affiliations:** ^1^Department of Biochemistry, Center of Immunity and Infection Lausanne, University of LausanneEpalinges, Switzerland; ^2^Genomics Platform, NCCR “Frontiers in Genetics,” University of Geneva/CMUGeneva, Switzerland; ^3^Functional Genomics Core at Nestle Institute of Health SciencesLausanne, Switzerland; ^4^Department of Proteomics and Signal Transduction, Max-Planck-Institute of BiochemistryMunich, Germany; ^5^Department of Pathology and Immunology, Faculty of Medicine, University of GenevaGeneva, Switzerland

**Keywords:** dendritic cell line, *in vitro* research

## Abstract

Research *in vitro* facilitates discovery, screening, and pilot experiments, often preceding research *in vivo*. Several technical difficulties render Dendritic Cell (DC) research particularly challenging, including the low frequency of DC *in vivo*, thorough isolation requirements, and the vulnerability of DC *ex vivo*. Critically, there is not as yet a widely accepted human or murine DC line and *in vitro* systems of DC research are limited. In this study, we report the generation of new murine DC lines, named MutuDC, originating from cultures of splenic CD8α conventional DC (cDC) tumors. By direct comparison to normal WT splenic cDC subsets, we describe the phenotypic and functional features of the MutuDC lines and show that they have retained all the major features of their natural counterpart *in vivo*, the splenic CD8α cDC. These features include expression of surface markers Clec9A, DEC205, and CD24, positive response to TLR3 and TLR9 but not TLR7 stimuli, secretion of cytokines, and chemokines upon activation, as well as cross-presentation capacity. In addition to the close resemblance to normal splenic CD8α cDC, a major advantage is the ease of derivation and maintenance of the MutuDC lines, using standard culture medium and conditions, importantly without adding supplementary growth factors or maturation-inducing stimuli to the medium. Furthermore, genetically modified MutuDC lines have been successfully obtained either by lentiviral transduction or by culture of DC tumors originating from genetically modified mice. In view of the current lack of stable and functional DC lines, these novel murine DC lines have the potential to serve as an important auxiliary tool for DC research.

## Introduction

Dendritic cells (DC) are the most efficient group of antigen-presenting cells. As such, DC are highly specialized in the detection and phagocytosis of pathogens, the processing of antigens as well as costimulation and inflammatory signals in order to induce adequate T cell responses. The potential of DC to define the quality and extent of an adaptive immune response has attracted major interest in vaccine science, DC being key targets to fight infectious as well as cancer diseases (Steinman, 2008).

Dendritic cells detect microbial ligands via Pattern Recognition Receptors (PRR) such as Toll-like Receptors (TLR; Reis e Sousa, 2004). Upon encounter with microbes, DCs are strongly activated, characterized by the upregulation of co-stimulatory molecules (CD40, CD80, CD86) and the production of cytokines and chemokines. In order to monitor and react efficiently to a pathogenic challenge, DC form a complex and heterogeneous network in the organism (Shortman and Naik, 2007; Merad and Manz, 2009). Several DC subsets have been described in both humans and mice (Shortman and Liu, 2002), the latter being the animal model preferentially used and most accessible in the field. Migratory DC capture pathogens at the site of infection and rapidly reach the nearest draining lymph node for antigen presentation. Conventional tissue-resident DC (cDC) act as sentinels in secondary lymphoid organs and other tissues for antigen capture and presentation *in situ*. Other inflammatory DC may differentiate from blood-derived monocytes and infiltrate secondary organs and tissue during infection or inflammatory response. DC types may be further subdivided into different subsets and are identified according to the expression of surface markers. For instance in the mouse spleen, DC subtypes include plasmacytoid DC (B220^+^ CD11c^int^ GR1^−^), monocyte-derived DC (MoDC; B220^−^ CD11c^int^ GR1^±^), and cDC (B220^−^ CD11c^high^ GR1^−^). The latter are commonly divided into CD8α^+^ (CD11c^high^, B220^−^, DEC205^+^, CD24^high^, CD11b^−^) and CD8α^−^ (CD11c^high^, B220^−^, DEC205^−^, CD24^low^, CD11b^+^, CD172^+^, CD4^±^) cDC subsets. Interestingly, several lines of evidence support the notion of division of labor and cross-talk within the DC network; altogether, DC subsets display differences in the capacity to monitor tissue or circulate, the expression of PRRs, the production of cytokines, as well as antigen uptake and presentation mechanisms (Reis e Sousa, 2004; Villadangos and Schnorrer, 2007; Pulendran et al., 2008).

Further to the inherent complexity and heterogeneity of the DC system, a number of technical challenges have set a bottleneck to advances in DC research. First is the natural scarcity of DC *in vivo*, which not only reflects their functional potency, but also is a major limitation on the cellular material available for experimentation (Inaba et al., 2009). Secondly, isolated splenic cDC show dramatic activation and apoptosis in culture, clearly detectable after a few hours of incubation (Vremec et al., 2011). This greatly hampers experimental settings whenever relatively large quantities of cells or long incubation times are required.

In contrast to the B-cell and T cell fields, there is not as yet a DC line thoroughly characterized and widely accepted for *in vitro* research. In the mouse model, the DC culture system that has been widely used is Bone Marrow-derived DCs (BMDC), based on the differentiation of DC by treatment of BM progenitors with GM-CSF (and IL-4, depending on the protocol; Inaba et al., 2009). More recently, BMDC are also generated using Flt3L, obtaining a mixture of equivalents to both CD8α^+^ and CD8α^−^ cDC subsets and pDC (Naik et al., 2005). In human DC research, DC are similarly derived from the culture of peripheral blood mononuclear cells or CD14^+^ monocytes with GM-CSF/IL-4 (the MoDC system; Inaba et al., 2009). In both the mouse BMDC and the human MoDC systems, DC differentiation is driven *in vitro*, during 6–10 days, and is often followed by LPS treatment overnight to “mature” DC. These methods provide large quantities of DC, but require repetitive sacrifice of mice or human blood sampling, and are relatively tedious and time-consuming, as compared to the use of immortalized cell lines.

A limited number of DC lines have been described. These include the D1 cells, a growth factor-dependent immature DC line derived from mouse spleen DC, which can be “matured” with LPS (Winzler et al., 1997; Mortellaro et al., 2009). The generation of murine DC lines based on oncogene-driven immortalization has also been reported, including the SRDC line (Ruiz et al., 2005), the SVDC line (Ebihara et al., 2004), and the DC 2.4 cell line (Shen et al., 1997). In humans, DC lines can be generated from the culture of leukemic DC found in the blood of acute myeloid leukemia patients (Mohty et al., 2003). Other related human and murine model cell lines used in the DC field are Raw264.7 and J774 in mice, and THP-1, HL-60 and MUTZ-3 lines in humans (Santegoets et al., 2008; van Helden et al., 2008). Some of the issues generally encountered with these DC lines or model cell lines are the requirement of particular growth factors or conditions to maintain cultures, as well as concerns over their equivalence to natural DC counterparts *in vivo*. In the light of the technical difficulties encountered in the study of DC biology, DC lines that retain the major functions of DC (further reflecting different subsets) and that are easily maintained in culture are still long sought.

In recent years, we developed a transgenic mouse expressing the SV40LgT oncogene (with an eGFP reporter) under the CD11c promoter, as a model system for histiocytic disorders such as severe forms of multisystemic Langerhans cells histiocytosis (Steiner et al., 2008). These mice indeed display DC tumorigenesis, mainly in the spleen and liver, which affects in particular the CD8α cDC subset. In addition to the relevance to histiocytosis, using this model, it has been possible to derive several murine DC lines, originating from CD8α DC tumors primarily in spleen (therefore termed MutuDC for “murine tumor”). Importantly, DC tumor cells are not indefinitely viable directly *ex vivo* but can undergo immortalization *in vitro*. We now present the derivation procedure used to generate these immortalized MutuDC lines, followed by their thorough characterization by direct comparison to WT splenic cDC. We validate that MutuDC lines have retained the major features characteristic of their natural counterpart, the normal CD8α cDC subset. These include the response to particular TLR-Ls such as CpG (TLR9-L) and PolyIC (TLR3-L) but not R-848 (TLR7-L), IL-12 secretion and antigen cross-presentation capacity. We furthermore show that the MutuDC lines may be modified by lentiviral transduction or by crossing the CD11c:SV40LgT-transgenic mice to the genetic background of interest to obtain genetically modified MutuDC lines. Finally, we discuss that the ease of culture and manipulation of the MutuDC lines render them a potent auxiliary tool to support advances in DC research.

## Materials and Methods

### Mice and treatments

The MutuDC lines derived are listed in Table 1 and originated from spleen tumors in CD11c:SV40LgT-transgenic C57BL/6 mice (Steiner et al., 2008). Other mice used were females or males at least 8 weeks old and were WT (C57BL6), Rag2^−/−^γc^−/−^ (Mazurier et al., 1999), MHC-I-deficient Bm1 mutant (B6.C-H2^bm−1^; Wilson et al., 2006; Lin et al., 2008), and the TCR-transgenic OT-1 Rag2^−/−^, OT-2, and T1 mice.

**Table 1:** **List of MutuDC line types currently available or in the process of being generated (“in progress”)**.

MutuDC line	DC type	Origin (mouse)		Insert (lentivirus)	Status (reference)
		Genotype	Background		
WT	CD8α^+^	CD11c:LgT-transgenic and otherwise WT (Steiner et al., 2008)	C57BL6		Available (Fuertes Marraco et al., 2011)
1IFNR^−/−^	CD8α^+^	CD11c:LgT-Tg × Ifnar1(tm1.Agt)	C57BL6		Available (Fuertes Marraco et al., 2011)
TLR3^−/−^	CD8α^+^	CD11c:LgT-Tg × Tlr3(tm1.Flv)	C57BL6		Available (Fuertes Marraco et al., 2011)
TLR9^−/−^	CD8α^+^	CD11c:LgT-Tg × Tlr9(tm1.Aki)	C57BL6		Available
MAVS^−/−^	CD8α^+^	CD11c:LgT-Tg × CARDIF604Siec	C57BL6		Available (Fuertes Marraco et al., 2011)
NOX2^−/−^	CD8α^+^	CD11c:LgT-Tg × Cybb (tm1.Din)	C57BL6		Available
NIK^−/−^	CD8α^+^	CD11c:LgT-Tg × Map3k14(aly)	C57BL6		Available
H-2^g7^ (NOD)	CD8α^+^	CD11c:LgT-Tg × NOD (Shi.Ltj) back-cross	NOD		Available
H-2^d^	CD8α^+^	CD11c:LgT-Tg × Balb/c back-cross	BALB/c		In progress
H-2^k^	CD8α^+^	CD11c:LgT-Tg × C3H back-cross	C3H		In progress
K^b−/−^	CD8α^+^	CD11c:LgT-Tg × H–2Kb (tm1)	C57BL6		In progress
IRF8^−/−^	CD8α^−^ (CD11b^+^)	CD11c:LgT-Tg × Irf8(tm1.Hor)	C57BL6		Available
BATF3^−/−^	CD8α^−^	CD11c:LgT-Tg × Batf (tm1.1Kmm)	C57BL6		In progress
Langerin:Tg	CD8α^+^	Langerin:LgT-Tg and otherwise WT	C57BL6		In progress
CD80/CD86^−/−^	CD8α^+^	CD11c:LgT-Tg × CD80^−/−^ × CD86^−/−^	C57BL6		In progress
Bim^−/−^	CD8α^+^	CD11c:LgT-Tg × Bcl2l11(tm1.1Ast)	C57BL6		Available
Bid^−/−^	CD8α^+^	CD11c:LgT-Tg × Bid (tm1.1Ast)	C57BL6		In progress
Noxa^−/−^	CD8α^+^	CD11c:LgT-Tg × Pmaip1(tm1.1Ast)	C57BL6		In progress
Bim^−/−^ Bid^−/−^	CD8α^+^	CD11c:LgT-Tg × Bcl2l11(tm1.1Ast) × Bid (tm1.1Ast)	C57BL6		In progress
Bim^−/−^ Noxa^−/−^	CD8α^+^	CD11c:LgT-Tg × Bcl2l11(tm1.1Ast) × Pmaip1(tm1.1Ast)	C57BL6		In progress
Caspase-1^−/−^	CD8α^+^	CD11c:LgT-Tg x Caspase-1^−/−^	C57BL6		In progress
IFNβ^+^ 1IFNR^−/−^	CD8α^+^	CMV:IFNβ+; CD11c:LgT-Tg × Ifnar1(tm1.Agt)	C57BL6	IFNβ (NM_010510)	Available
Luciferase^+^	CD8α^+^	CMV:Luciferase+; CD11c:LgT-Tg	C57BL6	Luciferase (AB261982)	Available
Indo^+^	CD8α^+^	CMV:Indo+; CD11c:LgT-Tg	C57BL6	IDO (NM_008324.1)	In progress
Arginase^+^	CD8α^+^	CMV:Arginase+; CD11c:LgT-Tg	C57BL6	Arginase (NM_007482.2)	In progress
IL-10^+^	CD8α^+^	CMV:IL-10+; CD11c:LgT-Tg	C57BL6	IL-10 (NM_010548.1)	In progress
latent TGFβ^+^	CD8α^+^	CMV:latentTGFβ+; CD11c:LgT-Tg	C57BL6	latent TGFβ (NM_011577.1)	In progress
active TGFβ^+^	CD8α^+^	CMV:activeTGFβ+; CD11c:LgT-Tg	C57BL6	active TGFβ (mutant Cys^223 and 225^; Fowlis et al., 1996)	In progress
Activin A^+^	CD8α^+^	CMV:ActivinA+; CD11c:LgT-Tg	C57BL6	Activin A (NM_002192)	
CTLA-4^+^	CD8α^+^	CMV:CTLA-4+; CD11c:LgT-Tg	C57BL6	CTLA-4 (NM_009843)	In progress
B7-H1/PDL1^+^	CD8α^+^	CMV:B7-H1+; CD11c:LgT-Tg	C57BL6	B7-H1 (NM_021893)	In progress
B7-DC/PDL2^+^	CD8α^+^	CMV:B7-DC+; CD11c:LgT-Tg	C57BL6	B7-DC (NM_021396)	In progress
IL-2^+^	CD8α^+^	CMV:IL-2^+^ CD11c:LgT-Tg	C57BL6	Il-2 (NM 008366)	In progress
IL12^+^	CD8α^+^	CMV:IL-12^+^ α and β as single chain construct with linker; CD11c:LgT-Tg	C57BL6	IL12α and β Fc (NM_001159424) (NM 008352)	In progress
IL35^+^	CD8α^+^	CMV:IL-12α and CMV:EBI3^+^ as single chain construct with linker; CD11c:LgT-Tg	C57BL6	IL-12α and EBI3 (NM 015766)	In progress
IL-15^+^	CD8α^+^	CMV:IL-15^+^; CD11c:LgT-Tg	C57BL6	IL-15 (NM 008357)	In progress

### Mutu DC lines

The DC lines are named after “murine tumor” (Mutu) followed by the number of the mouse they originate from and the genetic modification if applicable.

#### Culture conditions

The cell lines were derived and kept in culture at 37°C in a humidified incubator with 5% CO2. The complete medium composition was IMDM-glutamax (GIBCO 31980) adjusted with NaHCO_3_ to 310 mOsm if required and supplemented with 8–10% heat inactivated FCS (tested for endotoxin toxicity toward DC cultures), 10 mM Hepes (GIBCO 15630), 50 μM β-mercaptoethanol (GIBCO 31350), and 50 U/mL of penicillin and 50 μg/mL streptomycin (GIBCO 15070). The medium was not supplemented with additional growth factors. MutuDC line cells were harvested by incubation in non-enzymatic, 5 mM EDTA-based cell dissociation buffer (5 mM EDTA in 20 mM Hepes-PBS).

#### Derivation method and maintenance

**Figure 1:**
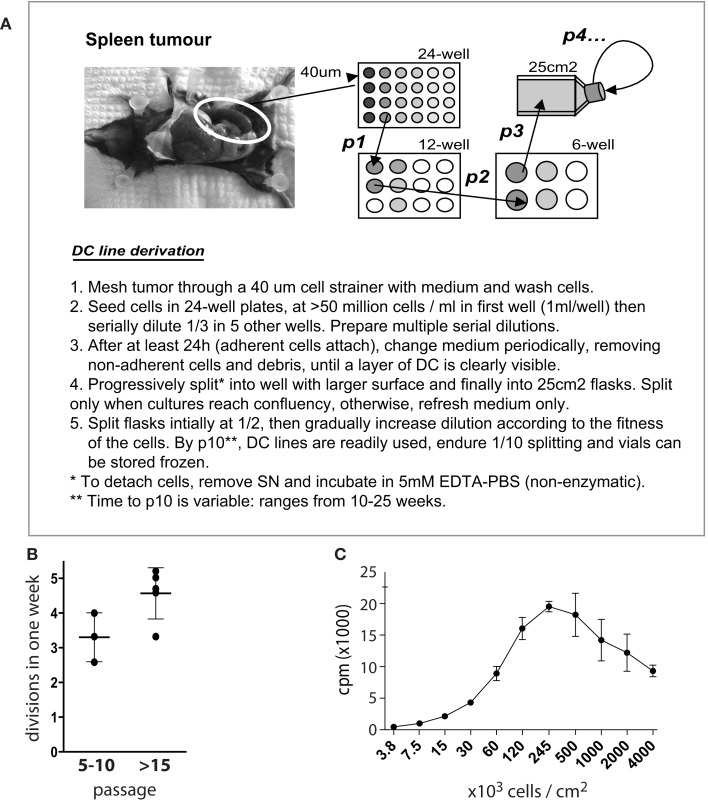
**Derivation of MutuDC lines**. **(A)** Schematic diagram and procedure for the derivation of MutuDC lines (NB. “*p*” followed by a number designates “passage”). **(B)** The number of cell divisions during 1 week is shown for eight different cell lines at different passages (*n* = 3 for passages 5–10, *n* = 5 for passage >15), based on trypan blue counts of MutuDC line cultures starting at 5 × 10^4^ cells per 0.5 mL per cm^2^. **(C)** The growth of one representative MutuDC line as a function of cell density is shown, measured by thymidine incorporation (*n* = 3) by seeding MutuDC line cells in flat-bottom 96-well plates at the indicated densities and adding 0.5 μCi of 3H-thymidine per well for 15 h. Data are presented as mean ± SD.

**Figure 2:**
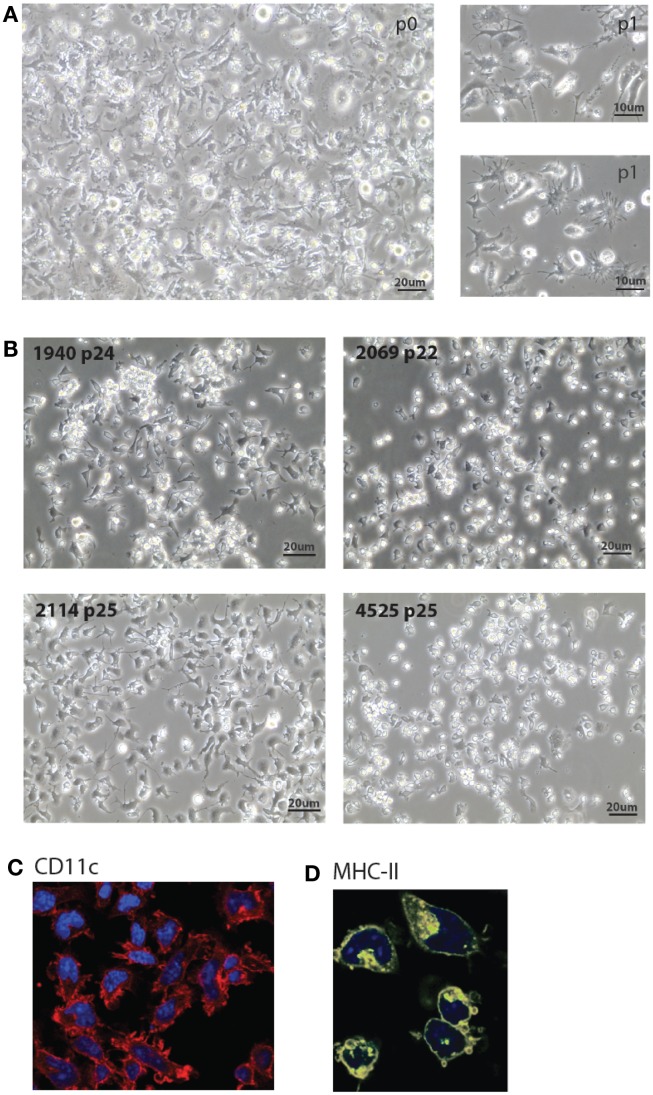
**Morphology of MutuDC line cultures**. **(A,B)**. Light microscopy images of five representative MutuDC lines at early [**(A)**, MutuDC line 4417] or later passages [**(B)**, MutuDC lines as indicated]. MutuDC lines may show different morphologies (in **(B)**], e.g., MutuDC1940 tends to show more aggregates and MutuDC2069 presents less dendrites. Moreover, these traits tend to be more pronounced in all cell lines with longer times of culture without splitting or harvest. **(C,D)** Immunofluorescence microscopy of one representative DC line (MutuDC2114) showing CD11c [in red, in **(C)**] and MHC-II [in yellow, in **(D)**]. Nuclei were stained with DAPI.

Figure 1 shows the standard procedure of MutuDC line derivation. Obtained from tumor-burdened animals, total splenocytes including tumoral DC are seeded in serial dilutions starting at high densities (>20 × 10^6^ cells per mL) in complete medium. The proportion of DC in tumors is variable, ranging from 10 to 50% of transformed CD8α DC. While the majority of cells (including DC) will die in culture, groups of adherent DC slowly appear in the cultures (Figure 2). Selection of the latter and removal of dead cells is allowed by periodic change of medium, until a monolayer of DC is clearly visible. Early passages are split at a maximum 1:2 dilution, and the dilution is progressively increased with passage. As a standard, MutuDC lines that reach passage 10 are normally capable of cell division at least once every 1.5 days (Figure 1B) and can be readily used. The derivation process up to passage 10 may last from 2 months to over half a year, but most MutuDC lines have been derived within 4 months. Established MutuDC lines (from passage 5 to 10 onward) are passaged by splitting at 1:10–1:15 from confluency, and re-seeded not lower than 25,000 cells per cm^2^ (Figure 1B). MutuDC lines can be kept in liquid nitrogen stocks (frozen at a density of 2–3 million per mL in 50% FCS and 10% DMSO in complete medium) and endure well such cryo-preservation. Use of the cells at passages higher than 50 is generally not recommended, as we have observed a clear decrease in their response and cytokine producing capacity above 70 passages (Figure A6 in Appendix). Of note, CD11c:LgT-Tg mice may develop DC tumors elsewhere than in spleen. The same DC line derivation procedure can be performed from DC tumors in thymus, liver, bone marrow, and lymph nodes (e.g., mesenteric and inguinal lymph nodes). Technically important to keep in mind, MutuDC lines are GFP positive due to the GFP reporter in the CD11c:SV40LgT transgene. Table 1 lists all the types of MutuDC lines that are currently available or in the process of being generated (“in progress”). The majority of experiments were done on the WT DC lines “MutuDC1940”, “MutuDC4525”, “MutuDC1995”, “MutuDC2069” and “MutuDC2114”. DC lines lacking genes of interest (“MutuDC*Ifnar1*^−/−^”, “MutuDC*Tlr3*^−/−^” and “MutuDC*Tlr9*^−/−^”) were generated by crossing the CD11c:SV40LgT-Tg mice (Steiner et al., 2008) to the relevant gene-targeted mice (Table 1).

### Splenic cDC subset purification

**Figure 3:**
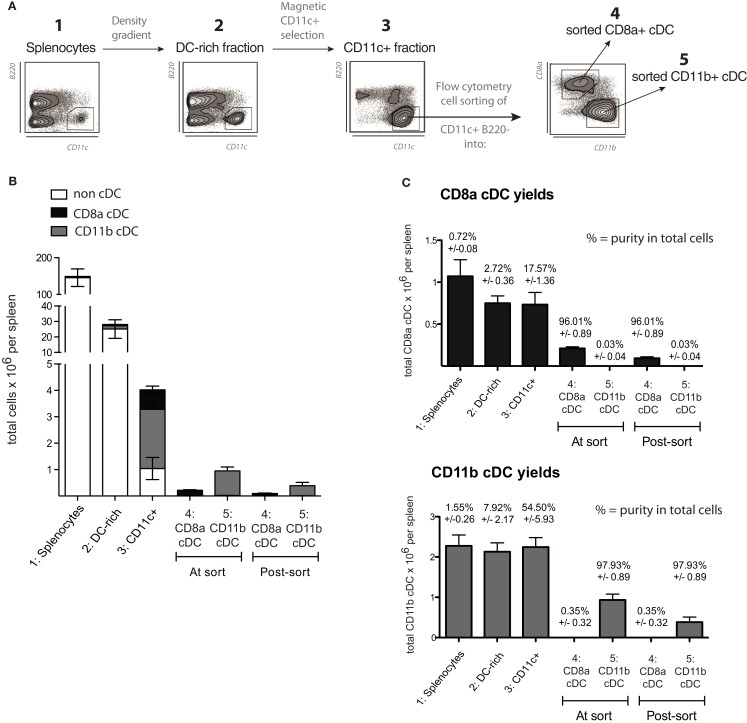
**Purification method and yields for the isolation of normal splenic cDC**. **(A)** Schematic diagram of the method used to purify cDC subsets from spleen, as detailed in Section “Materials and Methods.” A representative plot of B220 versus CD11c is given for steps 1–3, showing the increasing purity of splenic cDC (CD11c^+^ B220^−^ cells) throughout the procedure.**(B,C)** Yields based on total cell counts and purity for each splenic cDC subset in each sample throughout the isolation procedure, including the counts obtained during the cell sorting (indicated by the flow cytometry cell sorter) and the final yield based on sample counts after the cell sorting (*n* = 5). Data are presented as mean ± SD. **(B)** shows a vertically stacked bar graph of the yields to emphasize the very low relative frequency (and consequently yields) of splenic cDC subsets in total splenocytes. **(C)** Shows the yields and purities for each individual splenic cDC subset at each step of the isolation procedure. The values above each bar indicate purity ± SD.

Spleens were chopped and digested for 30 min at 25°C with collagenase D (1 mg/mL) and DNAse I (40 μg/mL) in RPMI-1640 complemented with 3% FCS, with occasional gentle mixing in order to extract and preserve maximal numbers of DCs. Cell suspensions were passed through 40 μm sieves and washed in PBS containing 5 mM EDTA and 5 μg/mL DNAse I and then resuspended in PBS containing 5 mM EDTA and 3% FCS. Total splenocytes were fractionated by density centrifugation in isohexol carbohydrate medium (Nycodenz, Axis-Shield, Norway) at 1.077 g/cm^3^ (Naik et al., 2006). The DC-enriched, light density fraction was collected and washed in PBS containing 5 mM EDTA prior to CD11c^high^ DC enrichment using anti-CD11c antibody-coupled magnetic micro-beads (Miltenyi Biotech). Alternatively, for proteomic experiments, CD8α^+^ cDCs were isolated as described previously (Luber et al., 2010). The cDCs represent 1–2% of total splenocytes. Within the cDC population, typically ∼20% were CD8α cDCs and ∼60% CD11b cDCs. Density centrifugation allowed for a ∼10-fold enrichment in DCs prior to immunomagnetic bead selection. Enriched DC preparations used for flow cytometry cell sorting contained more than 85% cDCs, and resulting cDC subsets were purified to at least 95%. At least 10 mice were required to isolate 10^6^ viable CD8α^+^ cDCs and 2.5 mice to isolate 10^6^ CD8α^−^ cDCs (Figure 3).

### Treatments *in vitro*

Dendritic cells stimulations were performed using PolyIC (5 μg/mL, Invivogen), CpG (2 mM, TriLink), R-848 (1 μg/mL, Invivogen), and LPS (5 μg/mL, Ultra-pure, Invivogen). Cross-linking CD40 antibody (clone FGK45, Miltenyi Biotech) was used at 10 μg/mL. IFNγ was used at 100 U/mL.

### Microscopy

#### Light microscopy

Cell cultures were directly photographed using the Leica Firecam 3.2 software on Leica DM IL LED microscope equipped with a Leica DFC295 camera.

#### Confocal microscopy

MutuDC lines were labeled with fluorochome-conjugated CD11c (clone N418, APC, Biolegend) and MHC-II (clone M5, APC, Biolegend). MutuDC lines were seeded on Poly l-lysine-coated slides overnight, fixed with 4% paraformaldehyde for 15 min at RT, and stained for 30 min at 4°C before mounting for fluorescence microscopy.

### Flow cytometry

The fluorochrome-conjugated antibodies used were specific to CD11c (clone N418, PECy7, eBioscience), CD45R-B220 (clone RA3-6B2, eF450, BioLegend), GR1 (clone RB6-8C5, PE, BioLegend, or PerCP-PECy5.5, eBioscience), CD8α (clone 54-6.7, FITC, eBioscience or APC-Cy7, BioLegend), DEC205 (clone 205yekta, PerCP-eF710, eBioscience), CD24 (clone M1/69, PerCP-Cy5.5, eBioscience), Clec9A (clone 42D2, PE, eBioscience), CD11b (clone M1/70, APC, eBioscience), SIRPα (CD172, clone p84, APC, Becton Dickinson), CD4 (clone RM4-5, APC, eBioscience), MHC-I (clone AF6-88.5.5.3, APC, eBioscience), MHC-II (clone M5/114.15.2, PE, Pharmingen), CD40 (clone 1C10, APC, eBioscience), CD80 (clone 16-10A1, PECy5, eBioscience), CD86 (clone GL1, APC, eBioscience, or Alexa750, BioLegend), and Va2 (B20.1, PE, eBioscience). Flow cytometric analyses were performed with FACScan, FACSCanto, or LSR II cytometers (Becton Dickinson) using FACSDiva (version 6.1.3, Becton Dickinson) and FlowJo (Version 9, Tree Star, Inc.) softwares for data processing.

### Gene expression analyses

#### RNA isolation and cDNA synthesis

Total RNA was isolated from MutuDC line samples and purified splenic cDC subsets using the spin columns from Qiagen RNeasy^®^ following the manufacturer’s protocol, with DNAse I digestion on-column and elution in 5 μL of RNAse-free water. Total RNA yields were quantified by Nanodrop spectrophotometry (Thermo Fisher Scientific, Inc.). The average yield was 5 μg/10^6^ cells for MutuDC lines and 200 ng/10^6^ cells for freshly isolated splenic subsets. Synthesis of cDNA was performed using random non-amer primers and the Superscript II Reverse Transcriptase kit (Invitrogen) starting from 0.2 to 1 μg of total RNA. Cleanup of cDNA was performed using spin columns from Wizard SV Gel and PCR cleanup (Promega), eluting cDNA in 50 μL of RNAse-free water and quantifying yield by Nanodrop as above. Samples were diluted at 3 ng/μL for Q-RT-PCR or at 50 ng/μL for semi-quantitative PCR, using 2 μL per PCR reaction (5 and 10 μL final volume, respectively).

#### Quantitative real-time PCR analysis

Quantitative real-time PCR (qRT-PCR) was performed using SYBR Green mix on LightCycler (capillary, 10 μL reaction) or LighCycler480 (384-well plate, 5 μL reaction) from Roche Diagnostics. The primers used for *Tlr3* and for *Tlr7* expression were as previously described (Edwards et al., 2003). Relative expression levels were analyzed using second derivative method with LC data analysis 3.5 Software (Roche). The housekeeping gene used was TBP. QRT-PCR was performed with at least technical duplicates. For the analysis, expression of each gene was normalized to the housekeeping gene, generating a single value per biological replicate.

#### Nanostring technology

Stimulations were performed in duplicate, using 500,000 cells per sample. Supernatants were collected for ELISA quantifications of IL12p40 and IL12p70 to confirm efficient activation (data not shown) while cells were collected for mRNA analyses using Nanostring technology. Cell lysis and RNA extraction were performed using spin columns from Qiagen RNeasy^®^ following the manufacturer’s protocol. RNA quality was controlled using an Agilent 2100 bioanalyzer and only samples showing minimal degradation (RIN > 8) were kept. RNA probes were ordered from NanoString^®^ Technologies and controlled for gene sequence specificity before use by qRT-PCR (data not shown). Hybridization and quantification was done according to the manufacturer’s protocol and quantification and normalization was performed using an *in-house* developed program running in a Microsoft Excel environment developed at the University of Geneva’s Genomics Platform.

### Cytokine secretion

Supernatants were assayed for IL-12 production by ELISA using the IL-12p40 and IL-12p70 kits from BD Biosciences according to the manufacturer’s protocol, except that the assay diluent was composed of 1% BSA and 10% FCS in PBS.

### Antigen presentation assays

Untouched OT-I CD8 T cells and OT-II CD4 T cells were purified from TCR-transgenic mice (OT-I and OT-II, respectively) by negative selection using immunomagnetic beads (Miltenyi Biotech). For direct MHC-I or MHC-II antigen presentation assays, MutuDC lines or freshly isolated DC were seeded at 10,000 cells per well in round-bottom 96-well plates. For MHC-I-restricted antigen presentation assays, DC were incubated for 2 h with SIINFEKL (OVA^257–264^) at the indicated concentrations, washed three times in medium, irradiated at 40 Gy, and incubated with 50,000 purified OT-I CD8 T cells (eFluor670-labeled). Similarly, for MHC-II-restricted antigen presentation, DC were pre-incubated for 2 h with OVA^323–339^ peptide, irradiated at 40 Gy, and incubated with 50,000 purified OT-II CD4 T cells (eFluor670-labeled). For cross-presentation of cell-associated antigen, MutuDC lines or freshly isolated DC were seeded at 25,000 cells per well in V-bottom 96-well plates, incubated with the indicated numbers of OVA-coated splenocytes (OCS, prepared from MHC-I-mutant Bm1 mice as previously described; Wilson et al., 2006), irradiated at 40 Gy, and incubated with 50,000 purified OT-I CD8 T cells (CFSE-labeled). T cell proliferation was measured after 60 h of culture by flow cytometry analysis excluding doublets and dead cells. OT-II CD4 T cells were gated as CD4^+^ Vα2^+^ cells and OT-I CD8 T cells were gated as CD8^+^ Vα2^+^ cells. Live dividing T cells were detected as low for cell proliferation dyes (eFluor670 low or CFSE low as indicated) and quantitated using calibration particles (BD Biosciences Pharmingen).

For H-2K^d^-restricted antigen presentation by MutuDC lines that were lentivirally transduced with H-2K^d^, the system used was the Plasmodium Berghei circumsporozoite (PbCs) peptide 252–260 (SYIPSAEKI) and the cognate T1 TCR-transgenic CD8 T cells as previously described (Segura et al., 2008). In round-bottom 96-well plates, 3,000 *K*^d^-positive or untransduced MutuDC line cells were seeded and pulsed with titrating concentrations of PbCs^252–260^ peptide for 2 h, washed three times in medium, irradiated with 40 Gy, and co-cultured with 10,000 CD8 T cells isolated from T1 TCR-transgenic mice (DC:T cell ratio = 1:3). Proliferation measured at day 3 by ^3^(H)-thymidine incorporation adding 0.5 μCi per well for the last 15 h of culture.

### Lentiviral transduction and transfection in MutuDC lines

Second generation lentiviral plasmids used were (pWP-SIN-cPPT-WPRE)-CMV-IRES-GFP lentiviral vector and the two packaging plasmids pMD2G and psPAX2. The lentiviral system and protocols have been described elsewhere (Salmon and Trono, 2007). Inserts (Table 1) were obtained by PCR amplification of cDNA from MutuDC line samples or splenocytes. Transduction was done into MutuDC lines, performing either mock (CMV:GFP; control) or specific (CMV:insert:GFP) transductions. The multiplicity of infection (MOI) used was between 5 and 10. Expression of the transgene began around day 4 post-transduction. As MutuDC lines already express GFP, only a shift in GFP was observed after transduction with GFP reporter-encoding transgenes. Efficiency of transduction was measured by FACS and WB for cellular proteins or ELISA for secreted proteins. For the representative lentiviral transduction shown in Figure 9A, the DC line “MutuDC1715 II” was transduced with H-2K^d^ as previously described (Segura et al., 2008). Table 1 lists other MutuDC lines modified by lentiviral transduction. Transfection with pDsRedExpress plasmid DNA (Clontech) was performed using the JetPEI solution (Chemie Brunschwig AG) according to the manufacturer’s manual or by a standard calcium phosphate transfection method. Electroporation was performed to transfect the pmaxGFP (Amaxa biosystems) with either Solution L or Solution V (Amaxa biosystems, Lonza) using the Amaxa Nucleofactor II device. pmaxGFP expression was distinguished from the endogeneous GFP by a shift in GFP expression level.

### Statistical analyses

Statistical analyses were performed using GraphPad Software Inc. Where indicated, *p*-values were obtained using two-tailed unpaired *t*-tests with 95% confidence intervals (ns = not significant; * = *p* < 0.05; ** = *p* < 0.01; *** = *p* < 0.001); Spearman’s rank correlation coefficients indicate 1 = perfect correlation, 0 = no correlation, −1 = perfect reverse correlation; Best-fit slope values were obtained by non-linear regressions using the robust fit method. Where shown, error bars indicate standard deviations.

## Results

### Derivation and maintenance of MutuDC lines

MutuDC lines are derived from CD8α^+^ DC tumors that develop in the previously described CD11c:SV40LgT-transgenic mice (Steiner et al., 2008). The derivation of MutuDC lines is based on the stabilization and growth of tumoral DC in culture, once transformation driven by the SV40 LgT oncogene has taken place *in vivo*. Figure 1 shows a diagram of the standard procedure for the derivation of MutuDC lines, further described in detail in Materials and Methods. Importantly, immortalization *in vitro* occurs “spontaneously” in standard complete medium, in absence of additional growth factors and with minimal manipulation. Resulting MutuDC lines may display different morphologies, ranging from growth exclusively in monolayers to a propensity to aggregate (Figure 2). This aggregation can be lost at later passages. As detailed in Section “Materials and Methods,” MutuDC lines from passage 10 onward divide at least once every 1.5 days (Figure 1B), can be stored frozen with optimal recovery upon thawing, and are easily maintained by standard cell culture practice. Growth is most efficient at a density of 2.5 × 10^5^ cells per cm^2^ (Figure 1C). For optimal maintenance, MutuDC line cultures should not be split lower than 5 × 10^4^ cells per cm^2^ (e.g., 10^6^ cells in one 25 cm^2^ flask). For standard experiments, MutuDC line cells are seeded at around 10^5^ cells per cm^2^, with variation depending on the kinetics of the experiment and considering the confluency at around 5 × 10^5^ cells per cm^2^.

### MutuDC lines versus purified splenic cDC *ex vivo*

In this study, we directly compared the MutuDC lines to splenic cDC *ex vivo*, in particular to their natural counterpart of origin, the CD8α^+^ cDC subset. It is important to note the technical difficulty that represents obtaining highly pure splenic cDC samples, and the limited viability of splenic cDC *ex vivo*. DC represent a minor fraction of immune cells, for example, out of a 100 million cells that can be easily recovered from a standard mouse spleen, as little as 2–3% are cDC (CD11c^+^B220^−^), of which typically 25% belong to the CD8α subtype (standard: 1 million spleen CD8α^+^ DC per mouse spleen). For our experiments, splenic cDC subsets were isolated by gentle collagenase digestion, followed by progressive enrichment in three steps (Figure 3): (1) Density gradient centrifugation to obtain a DC-rich fraction; (2) immunomagnetic bead-based enrichment of CD11c^+^ cells to purify cDC; and (3) flow cytometry-based cell sorting of cDC into highly purified cDC subsets (CD8α^+^ or CD8α^−^). An average of 200,000 CD8α^+^ and 900,000 CD8α^−^ (CD11b^+^) cDC could be recovered per spleen. Of note, due to stress, close to half of splenic cDC are lost immediately after flow cytometry sorting. Overall, this procedure allowed to minimize both costs and time, reducing cellular stress and optimizing survival of splenic cDC *ex vivo*, with DC yields in the expected range (Figure 3; Inaba et al., 2009).

Importantly, major advantages of MutuDC lines are their ease of maintenance in culture and expansion, being an unlimited and immediate source of material, as well as their high viability, as compared to the technical difficulties, workload and experimental limitations in using normal splenic cDC *ex vivo*.

### The MutuDC lines share characteristic surface markers and proteome profile with splenic CD8α cDC

**Figure 4:**
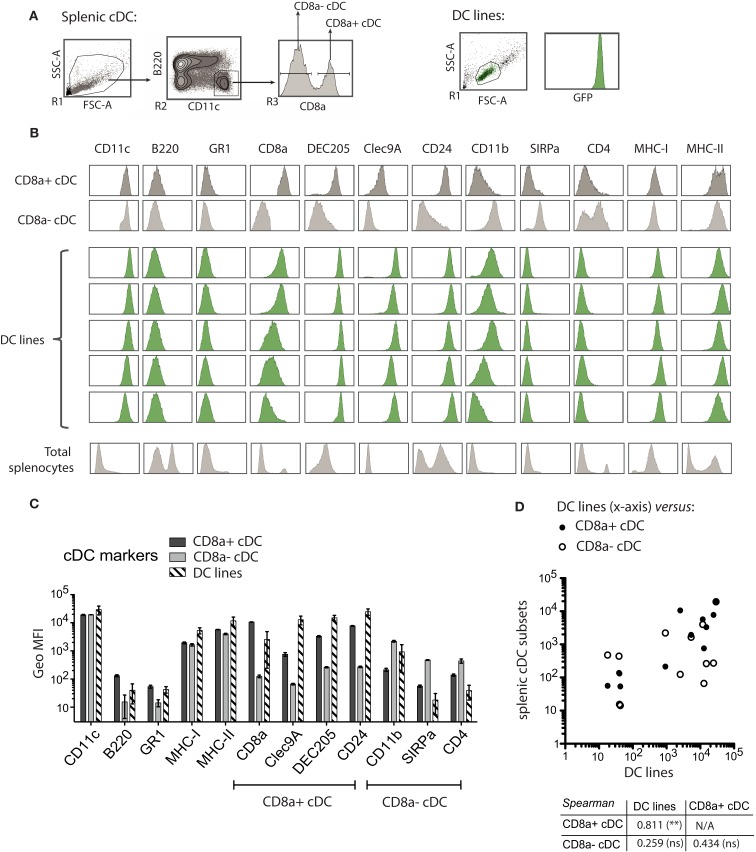
**MutuDC lines share the expression profile of surface markers with the splenic CD8α^+^ cDC subset**. **(A)** Gating strategy for the flow cytometry analysis of splenic cDC. MutuDC line cells (FSC versus SSC gate) were directly compared with splenic cDC. NB: MutuDC are eGFP positive. **(B,C)** The indicated surface markers were assessed on splenic cDC subsets (*n* = 2 per subset) and MutuDC lines (five independent DC lines). In **(B)**, histograms show one representative sample for splenic cDC in parallel to five representative MutuDC lines (from top to bottom: MutuDC4525, MutuDC1940, MutuDC2069, MutuDC2114, MutuDC1995). Histograms of total splenocytes are also shown as staining controls. In **(C)**, the geometric MFI is shown for the different surface markers as indicated for splenic cDC subsets (*n* = 2) and MutuDC lines (*n* = 5). Data are presented as mean ± SD. **(D)** Scatter plot and Spearman’s rank correlation coefficients based on the data shown in **(B)**. The scatter plot shows the average geometric MFI value of each surface marker and compares MutuDC lines (*x*-axis) versus the two splenic cDC subsets (*y*-axis) as indicated. Below the scatter plot, the Spearman’s rank correlation coefficient (and its *p*-value) comparing all pairs of DC types is shown.

**Figure A1:**
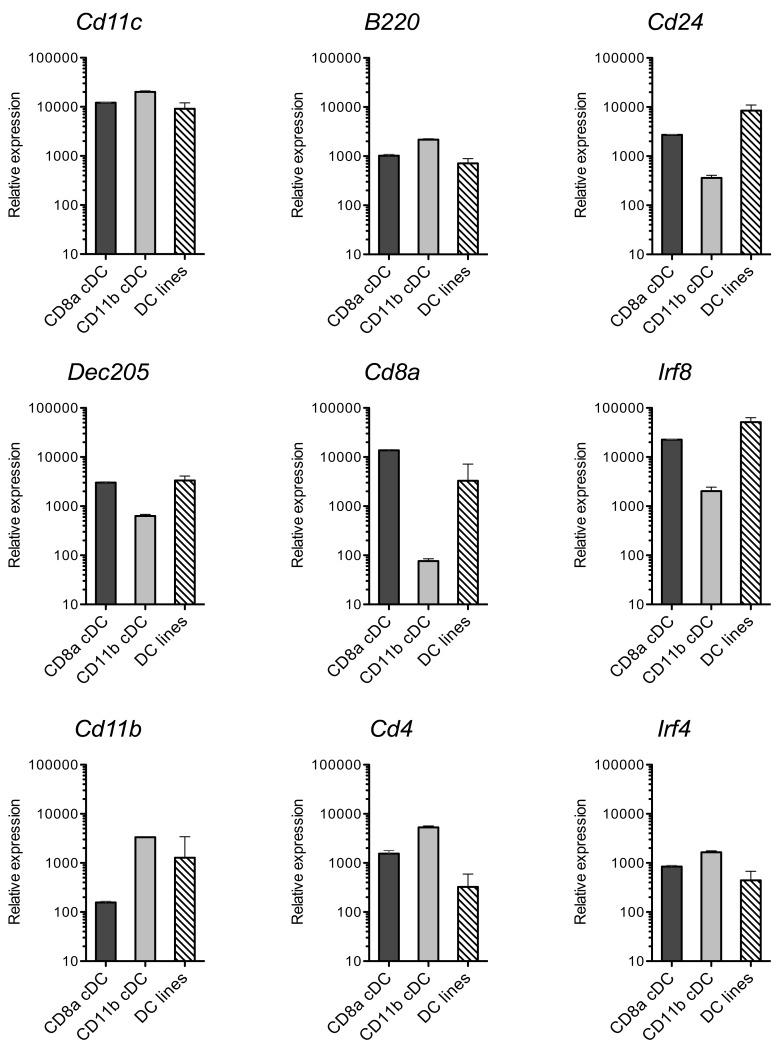
**MutuDC lines show a mRNA profile of DC markers similar to splenic CD8α^+^ cDC**. In complement to the analysis in Figure 4, levels of mRNA for the indicated genes were quantified by Nanostring technology in 13 different MutuDC lines and compared to freshly sorted splenic cDC subsets (*n* = 3 per subset). Error bars indicate SD.

The MutuDC lines derived were assessed for the expression of a panel of surface markers most commonly used to categorize DC subsets (Villadangos and Schnorrer, 2007), by direct comparison to normal WT splenic cDC subsets (Figure 4). These included markers characteristic of splenic CD8α^+^ cDC such as the endocytic receptor DEC205 and CD24 (Martinez del Hoyo et al., 2002; Naik et al., 2006) as well as the more recently discovered marker Clec9A (Caminschi et al., 2008; Huysamen et al., 2008; Sancho et al., 2008). Conversely, markers characteristic of CD8α^−^ cDC included CD11b, CD172, and CD4. Several of these markers were also assessed at the mRNA level, in parallel with the transcription factors IRF8 and IRF4 involved in CD8α^+^ and CD8α^−^ cDC subset development, respectively (Figure A1 in Appendix).

**Figure A2:**
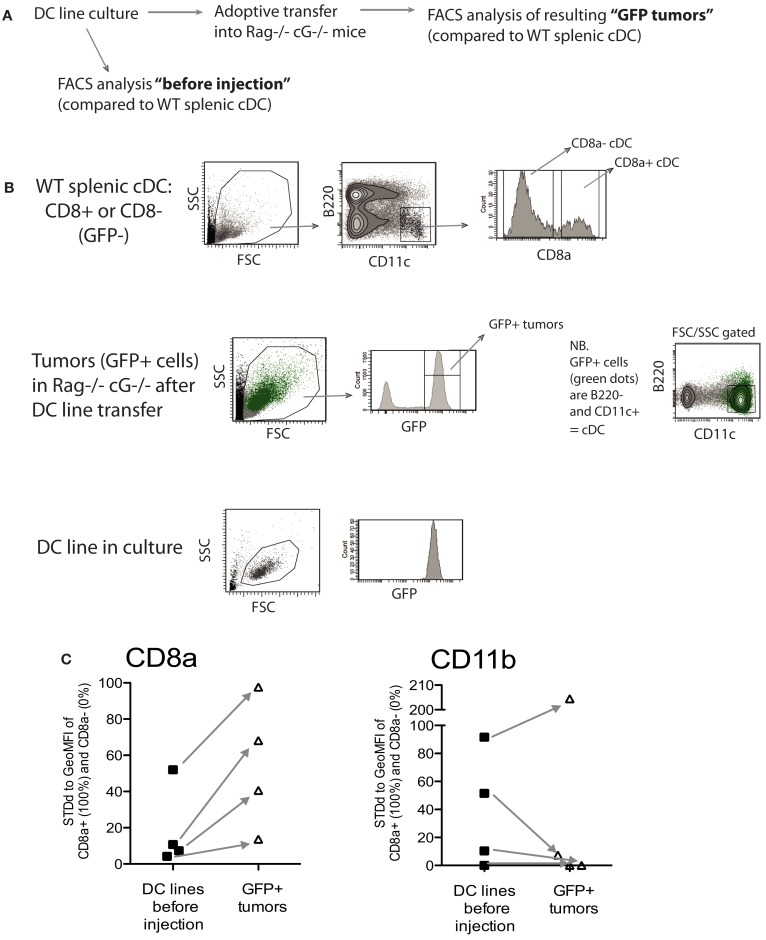
**Most MutuDC lines restore CD8α expression and downregulate CD11b upon adoptive transfer *in vivo***. **(A)** Experimental outline: MutuDC lines (*n* = 4) were analyzed directly from culture or following intra-veinous adoptive transfer of 3 × 10^6^ cells per MutuDC line into immunodeficient rag2^−/−^γc^−/−^ hosts. Spleens containing MutuDC line cells (tumors) were analyzed 16 days after injection. **(B)** Gating strategy for the flow cytometry analysis of MutuDC lines in culture versus tumors in MutuDC line-injected mice. MutuDC lines and normal WT splenic cDC subsets (used as controls) were gated as indicated (and as previously described in Figure 4A). MutuDC line cells in tumoral splenocytes from MutuDC line-injected mice were gated based on eGFP expression as indicated. **(C)** The expression of surface markers CD8α and CD11b was analyzed in MutuDC lines before injection versus following adoptive transfer *in vivo* and standardized relative to the levels found in normal WT splenic cDC subsets (controls). For CD8α expression, the CD8α^+^ (CD11b^−^) cDC subset represents 100% and the CD8α^−^ (CD11b^+^) cDC subset represents 0%, and vice versa for CD11b expression. Arrows indicate the change in surface marker expression for each particular MutuDC line.

As shown in Figure 4, MutuDC lines show surface markers of cDC: CD11c^high^ B220^−^ GR1^−^ and MHC-II^+^. Importantly, MutuDC lines closely resembled the CD8α^+^ subset and were positive for DEC205, CD24, and Clec9A while negative for CD4 and CD172 (Figure 4; Figure A1 in Appendix). At the mRNA level, MutuDC lines showed the differentially high expression of IRF8 and low expression of IRF4 characteristic of the CD8α^+^ cDC subset (Figure A1 in Appendix). Notably, different MutuDC lines showed variability in CD8α and CD11b levels, in some cases showing downregulation of CD8α and upregulation of CD11b as compared to splenic CD8α^+^ cDC (Figures 4B,C; Figure A1 in Appendix). This is possibly due to cell culture conditions, as MutuDC lines could regain CD8α expression and downregulate CD11b upon adoptive transfer *in vivo* (Figure A2 in Appendix). Pertinently, lack of CD8α expression has been observed in the BM-DC culture system, where CD24 or SIRPa are used as surrogate markers to identify CD8α^+^ or CD8α^−^ DC equivalents, respectively (Naik et al., 2005). In addition, while the function of CD8α and CD11b in DC is not known, human DC do not express CD8α and the equivalent of mouse splenic CD8α^+^ cDC has been recently identified as Clec9a^+^ DC in humans (Poulin et al., 2010; Villadangos and Shortman, 2010).

Overall, the pattern of surface markers in MutuDC lines closely correlated with splenic CD8α^+^, with a Spearman rank correlation coefficient of 0.811 comparing MutuDC lines and splenic CD8α^+^ cDC, versus 0.256 comparing MutuDC lines and splenic CD8α^−^ cDC (Figure 4D).

**Figure A3:**
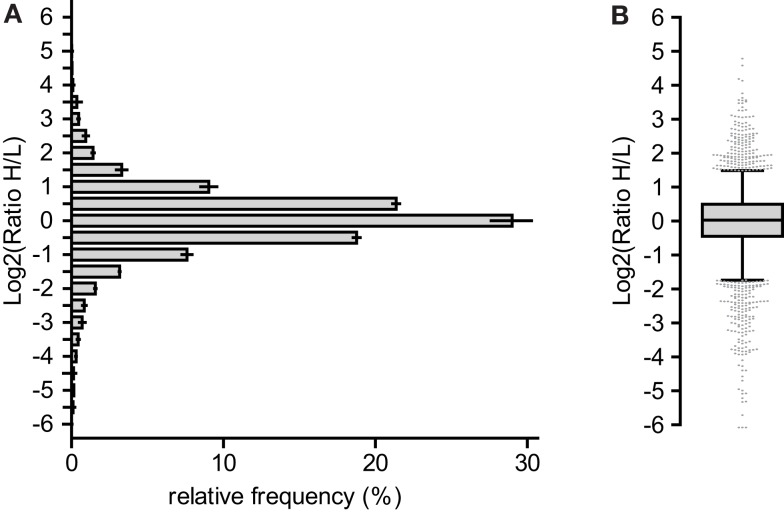
**Proteome analysis of DC line MutuDC1940 versus splenic CD8α^+^ cDC**. Lysates of the DC line MutuDC1940 and freshly isolated splenic CD8α^+^ cDC were analyzed as detailed in Appendix and previously described (Luber et al., 2010). The MutuDC1940 was SILAC-labeled and mixed 1:1 with primary CD8α^+^ cDCs. **(A)** Histogram of the frequency distribution of ratios from MutuDC1940 (H) versus *ex vivo* CD8α^+^ cDCs (L). Center of the bins are indicated. **(B)** Ratios of all quantified protein as boxplot with median and whiskers from 5 to 95 percentile.

Next, we compared freshly isolated CD8α^+^ DC from mouse spleens to our DC cell line on the proteome level. To achieve highest possible quantitation accuracy, we labeled MutuDC1940 cells with stable isotopes using SILAC and spiked it into *ex vivo* isolated CD8α^+^ DCs. Duplicate analysis revealed an unimodal distribution of ratios with 90% of the quantified proteins within a 2.1-fold expression difference between the MutuDC1940 and the *ex vivo* isolated CD8α^+^ cells (Figure A3 in Appendix, Proteomics.xls). These results indicate that the expression patterns of MutuDC lines and *ex vivo* isolated CD8α^+^ are very similar making the former an appropriate *in vitro* counterpart of CD8α^+^ DCs.

### The MutuDC lines have retained the functional characteristics of wild type splenic CD8α cDC

It was critical to assess if the MutuDC lines retained the major functional properties characteristic of splenic CD8α^+^ cDC. Compared to the CD8α^−^ subset, CD8α^+^ DC express higher levels of TLR3 and lack TLR7 expression (Edwards et al., 2003; Luber et al., 2010). In response to PAMP stimulation, CD8α^+^ DC are particularly capable of producing Th1 cytokines such as IL-12. Furthermore, distinct antigen presentation capacities have been attributed to different DC subsets, with CD8α^+^ DC being uniquely capable of antigen cross-presentation (Hildner et al., 2008). Altogether, CD8α^+^ DC are critical inducers of Th1 and CTL responses.

In order to assess the functional competence of MutuDC lines, normal WT splenic CD8α^+^ or CD8α^−^ cDC subsets were freshly purified and compared to MutuDC lines in the following functional assays *in vitro*.

#### MutuDC lines respond to TLR3 but not TLR7 stimuli

**Figure 5:**
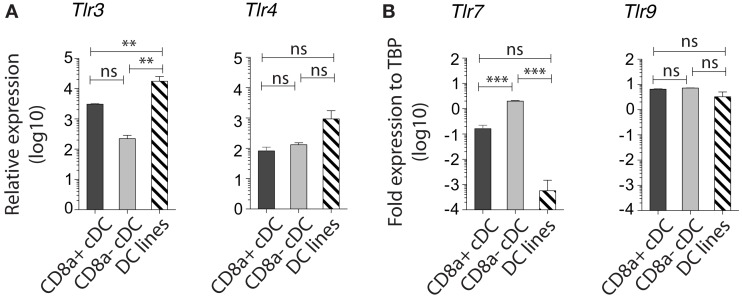
**MutuDC lines express TLR3 and lack TLR7, a characteristic of CD8α^+^ cDC**. Gene expression was analyzed by Nanostring technology for TLR3 and TLR4 **(A)** (*n* = 3 for splenic cDC subsets and *n* = 13 for MutuDC lines) and by quantitative RT-PCR for TLR7 and TLR9 **(B)** (*n* = 2 per splenic cDC subsets and *n* = 3 for MutuDC lines). Data are presented as mean ± SD. *p*-values were obtained by One-way ANOVA with Bonferroni post-test comparing all three DC types for each TLR individually (NB. The scale is logarithmic).

**Figure 6:**
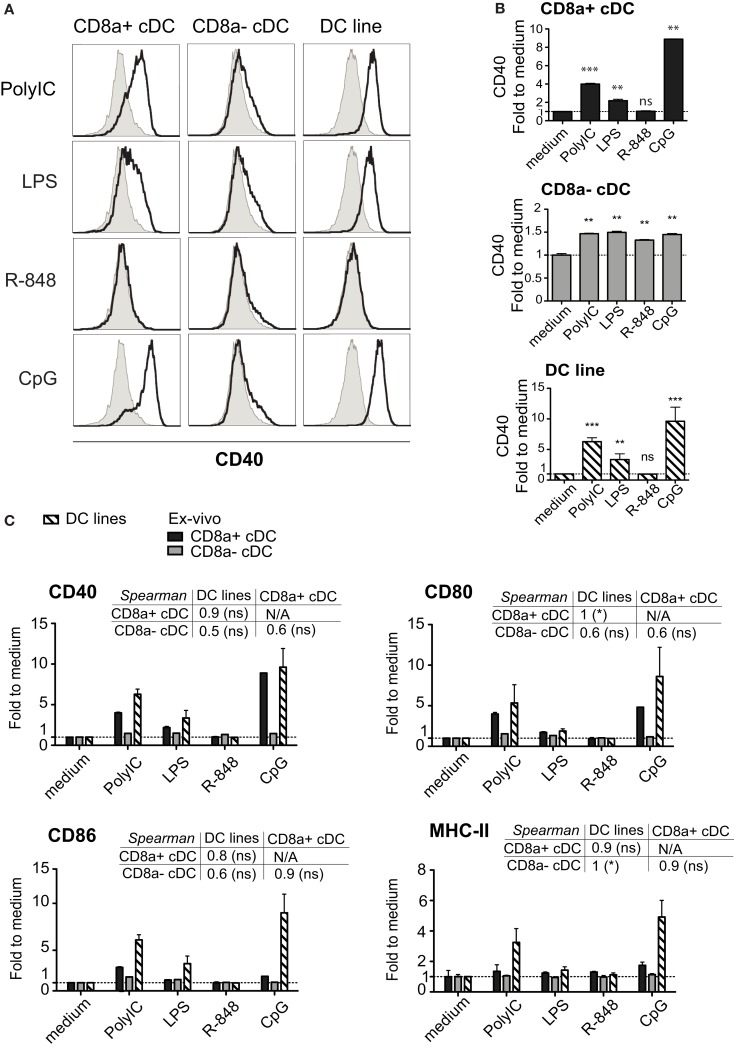
**The activation profile of DC lines in response to TLR stimulation is characteristic of splenic CD8α^+^ cDC**. DC lines (*n* = 4) and purified splenic cDC subsets (*n* = 2 per subset) were stimulated for 15 h with different TLR-Ls as indicated and analyzed for activation markers MHC-II, CD40, CD80, CD86. (**A)** Histograms show CD40 stainings for each stimulation (open) compared to medium (filled, gray). One representative sample is shown per DC type. **(B)** The geometric MFI of CD40 per treatment is shown individually for each DC type. **(C)**. The geometric MFI of CD40, CD80, CD86, and MHC-II per treatment is shown for the three DC types, with the corresponding Spearman’s rank correlation (and its *p*-value) coefficient comparing all three types of DC. Data are presented as mean ± SD.

Similar to splenic CD8^+^ cDC, the MutuDC lines expressed TLR3, TLR4, and TLR9, while showed no expression of TLR7, which was only detectable in CD8α^−^ cDC (Figure 5; Edwards et al., 2003). Accordingly, upon stimulation with the respective TLR-Ls *in vitro*, MutuDC lines strongly responded to PolyIC (TLR3-L), CpG (TLR9-L), and to a lesser extent to LPS (TLR4-L), by upregulation of co-stimulatory molecules CD40, CD80, and CD86 (Figure 6). Conversely, MutuDC lines showed no response to R-848 (TLR7-L). Such profile of TLR-L response in MutuDC lines corresponded to the responses observed in wild type CD8α^+^ cDC, and contrasted with the generally weaker activation in CD8^−^ DC, as supported by the higher Spearman rank correlation coefficients between MutuDC lines and CD8α^+^ cDC as compared to MutuDC lines versus CD8α^−^ cDC. The latter uniquely responded to TLR7-L by CD40 upregulation (Edwards et al., 2003). In response to TLR-Ls, the MutuDC lines also upregulated the activation marker CD70 (data not shown).

#### MutuDC lines efficiently produce the Th1-inducing cytokine IL-12 in synergistic response to TLR-Ls, IFNγ, and anti-CD40 stimuli

IL-12 production is one of the major features of splenic CD8α cDC (Hochrein et al., 2001), with its consequent Th1-polarizing and CTL-inducing function. Multiple signals are however required to fully activate IL12 production in DC, with particular combinations and time-points of stimulation resulting in different extents of IL-12 secretion (Macagno et al., 2007). For instance, microbial stimulation primes DC for subsequent CD40-induced IL-12 production (Schulz et al., 2000), in line with the synergies that have been observed amongst CD40, cytokine, and TLR stimulations (Snijders et al., 1998; Bosisio et al., 2002; Napolitani et al., 2005).

**Figure 7:**
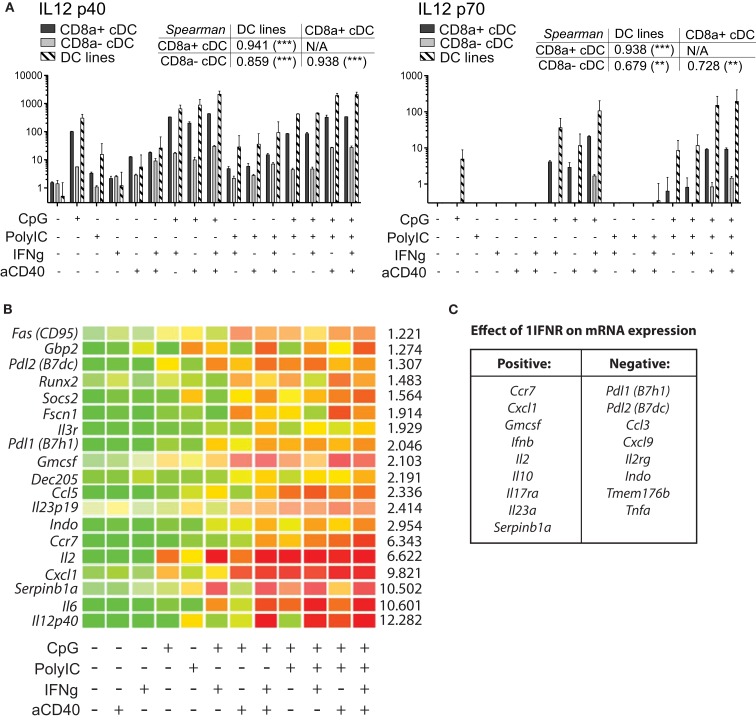
**MutuDC lines efficiently produce IL-12 p70 and show synergism in response to different combinations of TLR-Ls, IFNγ and aCD40**. **(A)** DC lines (*n* = 4) and purified splenic cDC subsets (*n* = 2 per subset) were stimulated with different combinations of TLR-Ls (CpG, PolyIC), IFNγ, and cross-linking anti-CD40, as indicated. The supernatants were collected after 15 h and analyzed for IL-12 p40 and p70 secretion by ELISA. Data are presented as mean ± SD, with the corresponding Spearman’s rank correlation (and its *p*-value) coefficient comparing all three types of DC. **(B)** MutuDC lines (*n* = 3) were stimulated for 20 h with different combinations of TLR-Ls (CpG or PolyIC), IFNγ, and cross-linking anti-CD40, as indicated, and mRNA expression was quantified by Nanostring technology. Genes shown in the heat map displayed a clear synergistic effect upon stimulation with multiple agents (green: low expression, to red: high expression). For each gene, a value for the synergistic increase is given (right side of the gene row) based on the fold increase in the stimulation with the full combination of reagents (CpG, PolyIC, IFNγ, and aCD40) *versus* the sum of stimulations with each reagent individually (Equation = value for “CpG, PolyIC, IFNγ, aCD40” divided by the sum of values for “CpG” + “PolyIC” + IFNγ + “aCD40,” with background subtraction for each value). **(C)** Impact of the lack of 1IFNR on mRNA expression of several genes upon stimulation. DC lines genetically deficient for the 1IFNR (*n* = 2 per treatment; derived from DC tumors in 1IFNR^−/−^ mice) were stimulated and analyzed as in, **(B)** compared to WT MutuDC lines. The gene expression data for eight representative genes upon stimulation in WT versus 1IFNR^−/−^ DC lines are shown in more detail in Figure A5 in Appendix.

In order to assess the capacity of MutuDC lines to produce cytokines, MutuDC lines and purified wild type splenic cDC subsets were stimulated with several combinations of TLR-Ls (CpG and/or PolyIC), IFN-g and cross-linking anti-CD40 antibody were assayed for IL-6 and IL-12 (p40 and p70) secretion (Figure 7A). TLR-Ls alone could induce substantial secretion of IL-12 p40 in both MutuDC lines and splenic CD8α^+^ DC, while CD8α^−^ DC secreted lower amounts of p40. For secretion of the bioactive IL-12 p70 subunit, TLR-Ls displayed synergy with IFNγ and anti-CD40. Importantly, the MutuDC lines were capable of efficient IL-12p40/p70 secretion in a manner comparable or higher than the freshly isolated CD8α^+^ cDC counterpart, while CD8α^−^ cDC produced low levels of IL-12p70 only with the full combination of stimuli. A similar secretion profile was observed for IL-6, with MutuDC lines and CD8α^+^ DC producing higher amounts and displaying synergy amongst treatments, in contrast to the poor induction of IL-6 in CD8α^−^ DC (data not shown).

Taking advantage of the easy access to large numbers of cells and their fitness in culture, the MutuDC lines were used to perform large-scale mRNA analysis upon numerous stimulations using Nanostring technology. Similarly to the experiments above, MutuDC lines were stimulated with different combinations of PolyIC, CpG, IFNγ, and anti-CD40 for 20 h (Figure 7B). A total of 188 genes were analyzed, of which 16 showed clear synergistic effects in mRNA upregulation upon treatment with different combinations of stimuli as compared to individual stimulations. As expected, these genes included *Il6* and *Il12p40*, in agreement with the experiments above.

Interestingly, other genes showing synergy were *Ccr7*, *Pdl1 (B7h1)*, *Pdl2 (B7dc)*, *Cxcl1*, *Indo*, and *Il2*. The complete list of the 188 genes and mRNA expression upon stimulation is accessible as supplemental data (Nanostring.xls). Using ELISA and Luminex analyses, MutuDC lines also secreted IL-1α and β, IL-2, IL-10, TNFα, CCL3/MIP-1α, CCL4/MIP-1β, CCL5/RANTES, CXCL2/MIP-2, CXCL3/KC, CXCL9/MIG, CXCL10/IP10, CXCL11//I-TAC, CX3CL1/Fractalkine similar to their *in vivo* counterparts upon stimulation (data not shown).

#### The MutuDC lines are capable of MHC-I and MHC-II-restricted antigen presentation, including cross-presentation

**Figure 8:**
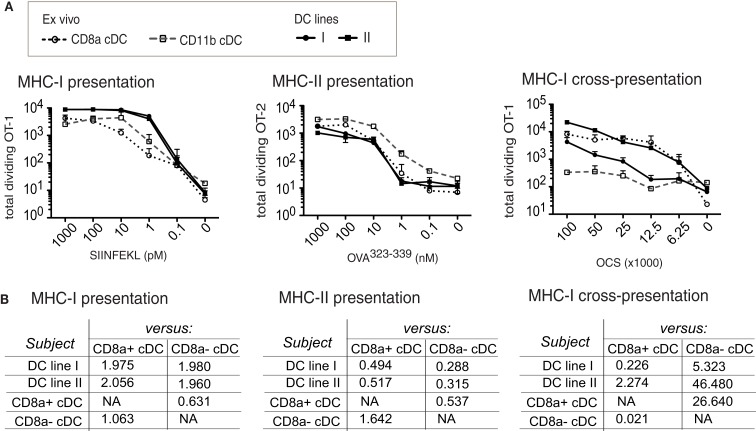
**MutuDC lines are competent for MHC-I- and MHC-II-restricted presentation to T cells, including cross-presentation, similarly to splenic CD8α cDC**. Two independent MutuDC lines (*n* = 2 per line) were directly compared to purified splenic cDC subsets for their capacity to present antigen to T cells**(A)**. Direct MHC-I-restricted antigen presentation: DC were loaded with SIINFEKL peptide at the indicated concentrations and co-cultured with OT-1-transgenic CD8 T cells. MHC-II-restricted antigen presentation: DC were incubated with Ova^323–339^ peptide at the indicated concentrations and co-cultured with OT-2-transgenic CD4 T cells. MHC-I-restricted cross-presentation: DC were co-cultured with ovalbumin-coated splenocytes (OCS) at the indicated numbers and further co-cultured with OT-1-transgenic CD8 T cells. T cell proliferation was quantified at day 3. The experiments are described in detail in Section “Materials and Methods.” Data are presented as mean ± SD **(B)**. Values of the best-fit slope comparing each DC type (“subject” column: MutuDC lines or splenic cDC subsets) to the splenic CD8α^+^ or CD8α^−^ cDC references (in columns, as indicated). The data from **(A)** were analyzed by performing non-linear fit regressions (with robust fit) as a measure of the relative magnitude of the response induced by different DC types. A best-fit slope of 1 means the subject (DC type, left column) and reference (“versus” columns: CD8α^+^ or CD8α^−^ cDC) are equally competent; >1 means the subject is more competent than the reference; <1 = subject is less competent than the reference.

While both CD8α^+^ and CD11α^−^ cDC subsets are capable of antigen presentation, CD8α cDC are particularly capable of cross-presentation of cell-associated antigens. Several antigen presentation modes were tested on MutuDC lines in comparison to normal splenic subsets, using the OT-I or OT-II TCR-transgenic T cell systems, including cross-presentation of cell-associated OVA to OT-I CD8 T cells (Wilson et al., 2006; Figure 8). While both splenic subsets were capable of direct presentation in both the MHC-I-restricted (SIINFEKL/OT-I) and the MHC-II-restricted (OVA323-339/OT-II) systems, CD8α^+^ cDC were remarkably more capable of antigen cross-presentation (Wilson et al., 2006; Figure 8). Albeit to varying degrees, MutuDC lines were capable of antigen presentation in all three settings (Figure 8A). Best-fit slopes were calculated to quantify the relative capacity to present antigen comparing the different DC types and, similar to splenic CD8α^+^ cDC, the MutuDC lines were particularly superior at antigen cross-presentation compared to CD8α^−^ cDC (best-fit slop of at least 5; Figure 8B). These results show that antigen-presenting capacity is preserved in MutuDC lines, notably the cross-presenting mode, similarly to their CD8α cDC counterparts.

### Generation of modified MutuDC lines

#### Lentiviral transduction of MutuDC lines

**Figure A4:**
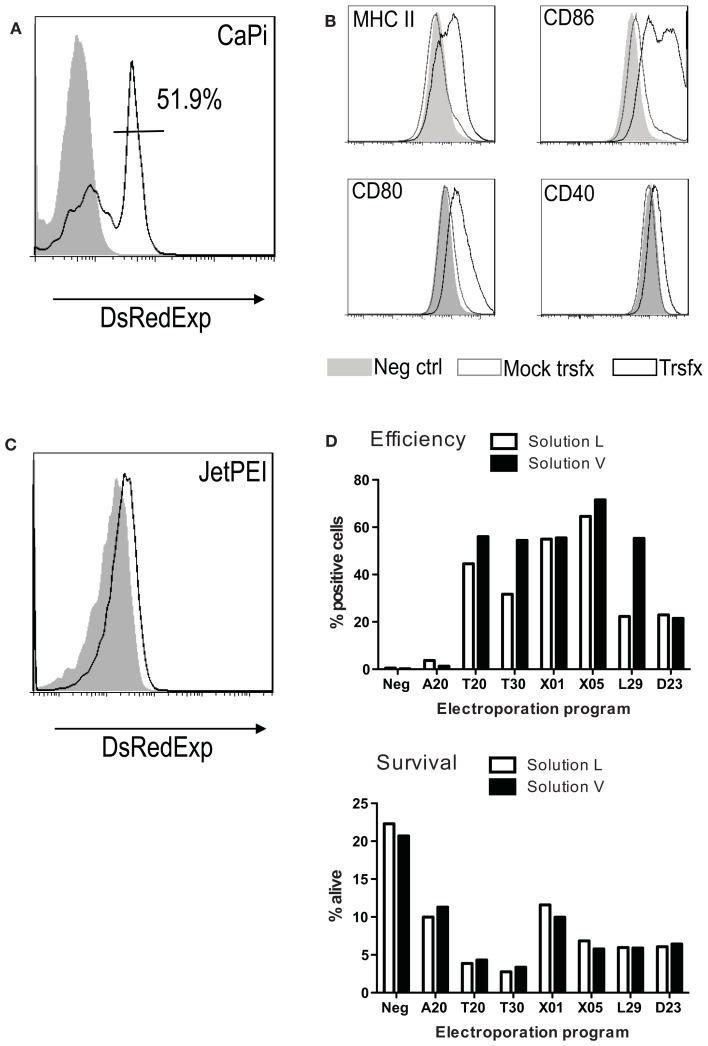
**Transfection and electroporation of MutuDC lines**. MutuDC line transfectability was tested using classical approaches such as calcium phosphate (CaPi) **(A,B)**, JetPEI **(C)** or electroporation **(D)**. The DsRedExpress **(A–C)** or pmaxGFP vectors **(D)** were used as test examples. CaPi transfection efficiency **(A)** and consequent upregulation of activation markers **(B)** 24 h post-transfection measured by flow cytometry (filled gray: negative control, open gray: mock transfection, open black: DsRedExpress transfected cells). **(C)** Transfection using JetPEI, with much weaker reporter gene expression as compared to CaPi in **(A)**. Electroporation efficiency and cell survival **(D)** at 24 h using several Amaxa electroporation programs with endotoxin free plasmid or electroporation solutions alone (Solution L and Solution V). In both electroporation and transfection (whether CaPi or JetPEI), all positive cells died within 72 h (data not shown).

The possibility to genetically manipulate cell lines is an important technical advantage. We tested several methods of gene over-expression in MutuDC lines. Transfection methods included the calcium phosphate-induced DNA precipitation method, the JetPEI PolyPlus transfection (Chemie Brunschwig AG) solution, and several protocols and solutions for the electroporation method (Amaxa biosystems, Lonza; Figure A4 in Appendix). While MutuDC lines showed transgene expression upon transfection using the calcium phosphate protocol and several electroporation methods (Figures A4A,D in Appendix), transfection activated the cells (Figure A4B in Appendix) and the viability of the cultures was greatly affected (Figure A4D in Appendix). Therefore, transfection methods only allowed short-time experiments.

**Figure 9:**
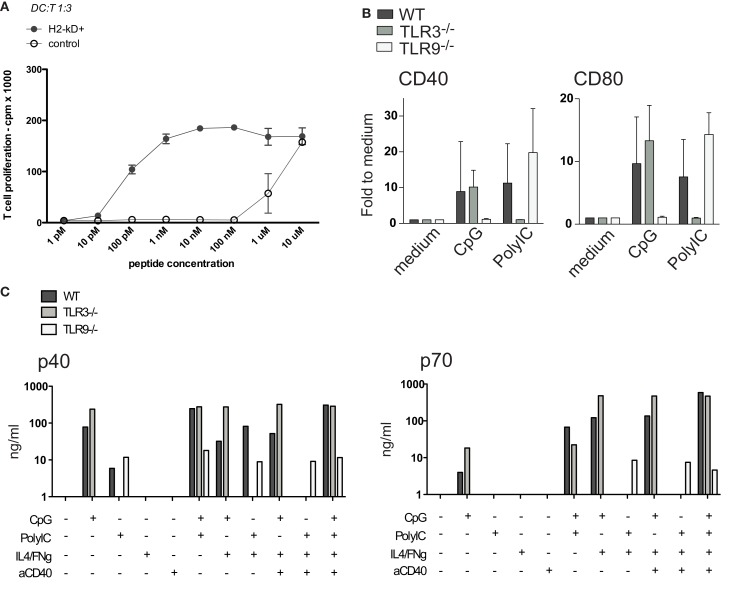
**Genetically modified MutuDC lines**. **(A)** One representative example of lentiviral transduction of MutuDC lines is shown, with H-2K^d^ over-expression and efficient induction of H-2K^d^-restricted antigen presentation. H-2K^d^-positive or control (untransduced) MutuDC lines were loaded with titrating amounts of the PbCs peptide as indicated and co-cultured with the relevant cognate T1 TCR-Tg CD8 T cells. **(B,C)** Representative genetically modified MutuDC lines are shown, deficient for *Tlr3* and *Tlr9*. WT (*n* = 3), *Tlr3*^−/−^ (*n* = 4) or *Tlr9*^−/−^ (*n* = 3) MutuDC lines were stimulated with PolyIC or CpG and analyzed after 18 h by FACS for the activation markers CD40 and CD80. **(B)** Similarly, the MutuDC lines were stimulated with different combinations of CpG, PolyIC, IFNγ, and aCD40 and analyzed after 18 h by ELISA on supernatants for IL-12 p40 and p70 secretion (*n* = 1) **(C)**.

In contrast, MutuDC lines could be successfully modified by lentiviral transduction. Figure 9A shows a representative example where MutuDC lines (C57BL6 background, H–2K^b^) could be modified to express the H-2K^d^ molecule and efficiently presented the *K*^d^-restricted peptide SIYPSAEKI to the cognate T1 TCR-Tg CD8 T cells.

#### Genetically knock-out MutuDC lines

Potentially, MutuDC lines can be derived from any knock-out or transgenic background of interest by crossing it with the CD11c-SV40LgT mice. As an example, the MutuDC*Tlr9*^−/−^ and MutuDC*Tlr3*^−/−^ DC lines were generated and as expected, did not respond to CpG and PolyIC, respectively (Figures 9B,C). In response to the relevant TLR-L, there is neither upregulation of co-stimulatory molecules (CD40, CD80; Figure 9B) nor secretion of IL-12 p40 (Figure 9C). Importantly, the synergies described above in IL-12 p70 secretion by TLRs/IFNγ/anti-CD40 combinations (Figure 7) are correspondingly lost in the relevant TLR knock-out MutuDC lines (Figure 9C).

**Figure A5:**
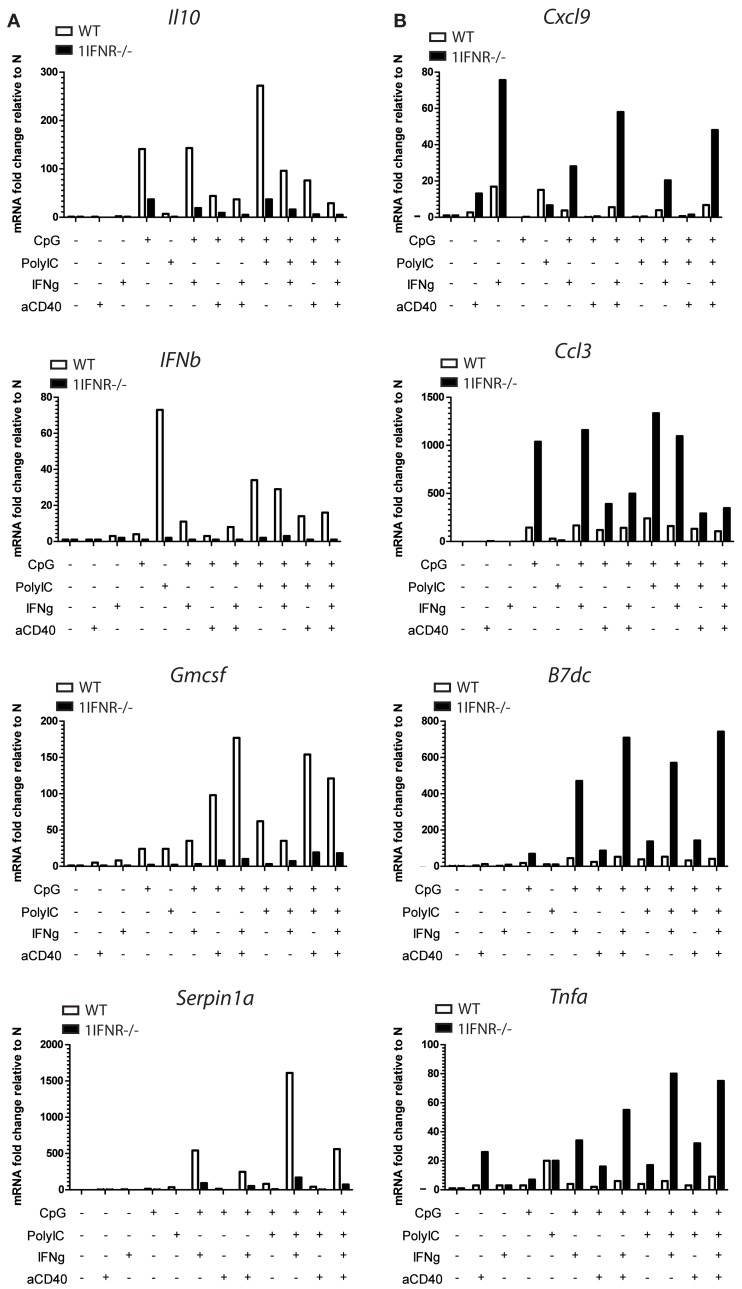
**Lack of the 1IFNR in MutuDC lines has a positive or negative impact on mRNA expression of several genes upon stimulation**. WT (*n* = 2) or 1IFNR (*n* = 2) MutuDC lines were stimulated for 20 h with different combinations of CpG, PolyIC, IFNγ, and aCD40 as indicated and described in Figure 7B, and analyzed by Nanostring technology. Figure 7C provides a summary table for the positive or negative effects of the 1IFNR on mRNA expression of several genes upon stimulation. Here, representative examples of positive **(A)** or negative **(B)** effects (four per group) are shown. Upon stimulation, the decrease in mRNA expression due to lack of 1IFNR (“positive” effect of 1IFNR) was 2- to 13-fold for *il10*, 3- to 17-fold for *Ifn*β, 5- to 20-fold for *Gmcsf* and 5- to 36-fold for *Serpinb1a*. On the other hand the lack of 1IFNR resulted in an increase in mRNA expression (“negative” effect of 1IFNR) that was 4- to 10-fold for *Cxcl9*, 3- to 7-fold increase for *Ccl3*, 3 to 18-fold for *B7dc* and 2- to 13-fold for *Tnfa*.

In addition, MutuDC lines lacking the *Ifnar1* (1IFNR^−/−^) were also generated and compared to WT MutuDC lines for gene expression in response to different combinatorial stimulations by Nanostring technology, as in Figure 7B. Interestingly, several genes were found to be differentially regulated upon stimulation in 1IFNR^−/−^ versus WT MutuDC lines (Figure A5 in Appendix), with positive and negative effects of the 1IFNR as summarized in Figure 7C. In absence of the 1IFNR, the upregulation of certain genes such *Tnfa* and *Pdl1* was enhanced, suggesting a negative regulatory effect of the 1IFNR. In contrast, other genes showed limited upregulation in 1IFNR^−/−^ MutuDC lines, suggesting a positive role of the 1IFNR on their expression. The latter included *Ifnb* as expected (positive regulatory effect of 1IFN on 1IFN production), as well as *Il10* and *Ccr7*.

Table 1 shows a list of the MutuDC lines that are currently available and in the process of being generated (“in progress”), including lentivirally transduced DC lines, or DC originating from genetically modified mice.

## Discussion

The technical difficulties to obtain substantial numbers of viable primary DC (Figure 3) are one major argument in favor of the use of DC lines in screening or preliminary experiments, prior to confirmation using DC *ex vivo* or *in vivo*. However, there is only a limited number of DC lines and DC culture systems described at present (van Helden et al., 2008; Inaba et al., 2009).

The MutuDC lines we here describe rely on cellular transformation driven by the SV40 large T oncogene. Strategies for the derivation of DC lines by oncogene-driven immortalization have been previously described, including the use of bone marrow from SV40-LgT-temperature sensitive-transgenic mice (SVDC line, derived with GM-CSF, at 33°C; Ebihara et al., 2004). The resulting SVDC line represents an enhancement to GM-CSF-driven BMDC given the relatively larger number of cells available, but the requirement of culture at 33°C may represent a technical limitation. A second cell line, the SRDC line, with a CD8a^+^ phenotype, results from transformation of splenic DC *in vitro* by transfection with a plasmid coding for SV40 LgT (Ruiz et al., 2005). A third cell line is the DC 2.4 cell line, based on supertransfection with myc and raf in GM-CSF-transduced BM cells (Shen et al., 1997). In contrast to previous studies, the MutuDC lines we describe result from transformation of DC *in vivo*. With expression of the SV40LgT oncogene under the CD11c promoter, transformation is not only primarily restricted to DC, but it occurs at the late stages of DC ontogeny when CD11c is highly expressed and in particular in the CD8a^+^ cDC subset (Steiner et al., 2008). Within the first days of culture, a large majority (ca. 90%) of DC tumor cells die, suggesting that DC tumor cells are not as yet immortal. Rather, immortalization is achieved by the spontaneous selection of the minority of DC tumor cells that stabilize their survival and growth in culture. Other non-oncogene-driven DC lines previously reported include the D1 cells, derived from mouse spleen DC under the strict supplementation of GM-CSF and other fibroblast-derived growth factors, that require maturation by LPS stimulation (Winzler et al., 1997; Mortellaro et al., 2009). Importantly, and also in contrast to previously described DC lines, the derivation of the MutuDC lines we report does not require any additional growth factors nor culture conditions (e.g., temperature/CO2), nor supplementary induction of maturation by LPS.

Given the complexity of the DC system, with phenotypic and functional heterogeneity amongst DC subsets, the equivalence of DC lines to a normal DC subset counterpart is crucial. We have addressed in detail the equivalence of the MutuDC lines by direct comparison to freshly isolated normal and WT splenic cDC subsets. Altogether, we show that the MutuDC lines have retained the major characteristics of the splenic CD8α^+^ cDC subset. These feature the expression of major surface markers characteristic of splenic CD8α^+^ cDC (DEC205, CD24, and Clec9a, while no expression of CD4 and CD172), as well as upregulation of co-stimulatory markers in response to TLR-Ls, including a response to PolyIC (TLR3-L) and not to R-848 (TLR7-L), in agreement with their profile of TLR expression (TLR3+ TLR7-). The MutuDC lines showed comparable or superior secretion of IL-12. It is possible that the fitness of MutuDC lines in culture, compared to the rapid death of splenic cDC *ex vivo*, accounts at least in part with such superiority in MutuDC lines. In addition, MutuDC lines could efficiently present antigen in the context of both MHC-II and MHC-I, including direct antigen presentation and the cross-presentation of cell-associated antigens.

**Figure A6:**
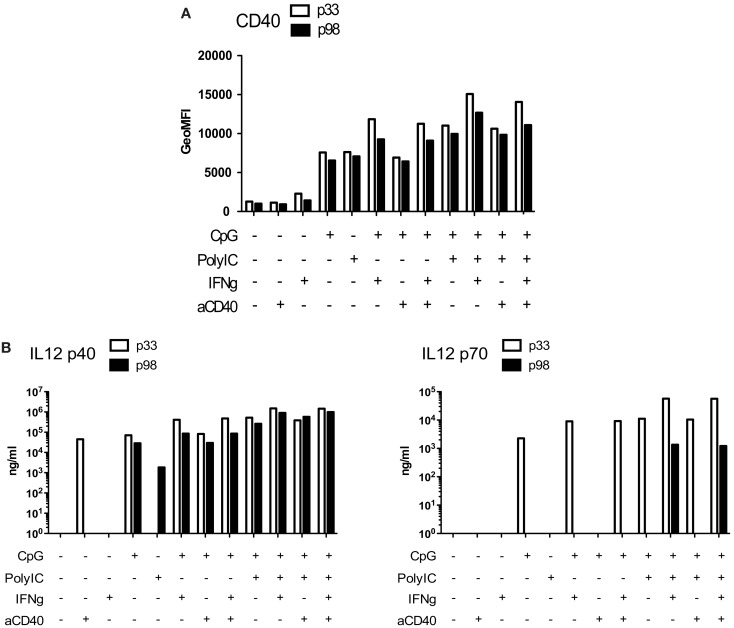
**MutuDC lines keep their ability to upregulate activation markers upon stimulation even at high passage numbers**. **(A)** Cells with low (33, open bars) or high (98, black bars) passage number were stimulated for 20 h with different combinations of CpG, PolyIC, IFN-g, and aCD40 as indicated. Analysis of CD40 expression by flow cytometry is shown (similar results were obtained for CD80, CD86, and MHC-II, data not shown). **(B)** MutuDC lines at p33 or p98 were stimulated as indicated [similar to **(A)**] and were analyzed for IL-12 p40 and p70 secretion in the cell culture supernatants.

Once established, the MutuDC lines are readily cultured for months, representing a potent DC research tool, being possible to readily obtain millions of cells that survive and continue growing in normal culture conditions. The upregulation of activation markers and IL-12 p40 secretion remained comparable between passages 33 and 98, that is, following at least 12 months in culture (Figures A6A,B in Appendix). In contrast, the capacity to secrete IL-12 p70 was severely diminished at p98 (Figure A6B in Appendix). The MutuDC lines are therefore generally not used after around 50 passages. Nevertheless, the capacity to produce IL-12p70 could be restored upon adoptive transfer of MutuDC line cells *in vivo* (data not shown).

The availability of the MutuDC line cells is in remarkable contrast to the scarcity, workload and costs that obtaining one million CD8α cDC demands, added to the few hours survival of the freshly isolated cDC in culture. Further to the culture of millions of DC without a timeframe limitation, perhaps the most outstanding advantage of the MutuDC line system is the number of mice that it can replace, potentially being in the order of billions of mice. Based on the final yield (Figures 3B,C), at least 10 mice were required to isolate 10^6^ viable CD8α^+^ cDCs and ca. 2.5 mice to isolate 10^6^ CD8α^−^ cDCs. This is valuable for the implementation of the 3R principle in animal experimentation, namely “Reduce, Refine, and Replace,” particularly relevant in the context of DC research, given the scarcity of tools for *in vitro* research and the difficulties in experimentation *in vivo*. For instance, in order to have enough mRNA material for the Nanostring hybridization that we performed, a total of 18 million cells was required to analyze the 12 different stimulations in duplicate (Figure 7). At our estimated 10^5^ CD8α^+^ cDC obtained per mouse, this requirement in cell numbers would have been equivalent to the use of at least 180 mice. In addition to the workload and costs of DC isolation, the DC need to maintain their fitness in culture during the 20 h stimulation (particularly for mRNA isolation), which is not possible using freshly isolated cDC. Clearly technically advantageous, with both excellent yield and viability, the MutuDC lines allow the performance of large-scale experiments that would otherwise be extremely difficult if not impossible using freshly isolated DC.

It has also been possible to generate several genetically modified MutuDC lines, either from breedings, crossing the CD11c:SV40LgT-TG mice to other genetic backgrounds of interest, or by lentiviral transduction of MutuDC lines (Table 1). Genetic backgrounds that alter DC ontogeny or homeostasis may potentially lead to the generation of DC tumors other than CD8α^+^ cDC tumors. A strategy to generate splenic CD11b MutuDC lines is currently underway, which consists in crossing the CD11c-SV40LgT-TG mice to BATF3-deficient mice, which lack the CD8α^+^ DC subset, potentially resulting in the development of DC tumors of the CD11b subtype.

In point of fact, MutuDC lines share with BM-DC many of their advantages over primary DC (*ex vivo*), such as the high numbers and the fitness of the cells that can be achieved. However, MutuDC lines remain more advantageous than BM-DC as they can be maintained in culture over months as opposed to the few days period where BM-DC can be used experimentally. Per mouse, the number of cells obtained is also much superior in MutuDC lines (expansion for over 50 passages once established) compared to the repetitive sacrifice of mice needed for BM-DC cultures (in the range of 10^7^–10^8^ cells obtained per mouse for the GM-CSF derivation and 10^6^–10^7^ for the Flt3L derivation).

Both MutuDC lines and BM-DC share concerns on their equivalence to normal DC subsets found *in vivo*. In contrast to the relatively heterogeneous DC that are obtained by BM differentiation in BM-DC, the MutuDC lines are homogeneous and resemble the CD8α^+^ subset. This is both an advantage and a limitation depending on the research interest. Noteworthy, the interest in the CD8α^+^ cDC subset has been exponentially increasing over recent years, with the identification of its functional equivalent in humans (Poulin et al., 2010; Villadangos and Shortman, 2010). This subset has unique capacities to cross-present cell-associated antigens and to induce Th1 and CTL responses, and is therefore of central interest in vaccine science and immunotherapy, including strategies to target antigens to the CD8α^+^ subset (Hildner et al., 2008; Shortman and Heath, 2010). At present, the majority of DC culture systems, including DC lines and BM-DC, rely on growth factors such as GM-CSF, which preferentially expand the CD11b^+^ CD24^−^ (CD8α^−^) subset of DC (Shen et al., 1997; Winzler et al., 1997; Ebihara et al., 2004; Naik et al., 2005; Mortellaro et al., 2009). In order to obtain DC cultures of the CD8α^+^ subset, DC are generally derived from BM using Flt3L rather than GM-CSF (Naik et al., 2005). Only one DC line with a CD8α^+^ phenotype has been previously reported, the SRDC line (without further follow-up studies; Ruiz et al., 2005).

The MutuDC lines we here report are therefore uniquely interesting as we validate that they have retained the major characteristics of the CD8aα^+^ cDC subset by direct comparison to their normal counterpart, they are readily manipulated and show excellent viability in culture. Overall, the MutuDC lines have the potential to serve as a powerful tool for screening and preliminary experimentation, in particular when the cross-presenting CD8α^+^ cDC subset is investigated. Artifacts arising due to the SV40LgT-driven tumorigenesis and immortalization of MutuDC lines cannot be excluded, and findings made in MutuDC lines need careful confirmation in normal DC and *in vivo*.

For instance, we have recently shown that Type I Interferon induces apoptosis preferentially in the splenic CD8α^+^ subset following PolyIC stimulation and could show the role of BH3-only proteins in this process *in vivo* (Fuertes Marraco et al., 2011). Notably, the MutuDC lines strongly assisted our studies: screening of TLR-Ls and kinetic studies in MutuDC lines preceded targeted experimentation *in vivo*. This was critical to choose parameters as simple as the range of time-points to be analyzed, but also to choose the genetic deficiencies that could confirm the proteins involved in the process by first screening for apoptotic proteins with western blot analyses in MutuDC lines. Conversely, following the observation that the 1IFNR was involved in splenic cDC depletion, the induction and role of IFNβ in DC apoptosis was more thoroughly investigated *in vitro* using the MutuDC lines. Notably, it was determined that TLR3 is necessary for induction of IFNβ in CD8α^+^ DC (in isolation, *in vitro*), and that exogenous IFNβ alone could induce apoptosis. The latter experiments were performed first using MutuDC lines, prior to the experiments on IFNβ production and induction of apoptosis using WT and PRR knock-out mice. Furthermore, the availability of 1IFNR^−/−^ MutuDC lines has also been of benefit for the in-house production of IFNβ and was used to prove that IFNβ alone was sufficient to induce MutuDC line apoptosis and splenic DC depletion.

Altogether, the effective strategy for DC research using MutuDC lines is a compromise between firstly, screening and performing as many experiments as needed in the MutuDC lines to acquire preliminary evidence on a given aspect of DC biology, and secondly, ultimate confirmation *in vivo*. The use of MutuDC lines has the potential to enormously expand the range of experimental parameters, conditions and biological candidates to be considered by allowing extensive preliminary experimentation, and subsequently greatly reduce the number of experiments, time, materials, and finally, mice that are needed for *in vivo* experimentation.

## Conflict of Interest Statement

The authors declare that the research was conducted in the absence of any commercial or financial relationships that could be construed as a potential conflict of interest.

## Supplementary Material

The Supplementary Material for this article can be found online at http://www.frontiersin.org/Antigen_Presenting_Cell_Biology/10.3389/fimmu.2012.00331/abstract

## Appendix

### Proteomics

#### Sample preparation

The MutuDC1940 was subjected to SILAC labeling, by culture in complete medium with natural arginine and lysine replaced by heavy isotope-labeled l-arginine-U-13C6-15N4 and l-lysine-U-13C6-15N2 (Cambridge Isotope Laboratories, Andover, MA, USA) as described previously (Ong et al., 2002; MCP 2002, 376-86). SILAC-labeled MutuDC1940 and *ex vivo*-purified CD8a+ DCs were washed in PBS and immediately frozen on dry ice. Cells were lysed in 2x LDS sample buffer (NuPAGE, Invitrogen), mixed 1:1 based on cell numbers, and separated by 1D-SDS PAGE (4–12% Bis-Tris Mini-Gel, Invitrogen). After Colloidal Blue Staining (Invitrogen), gel pieces were excised from the gel and subjected to reduction, alkylation, and in-gel digestion with sequence grade modified trypsin (Promega) as described (Shevchenko et al., 1996). After digestion, peptides were extracted by 30% acetonitrile in 3% TFA in water, reduced in a speedvac, and desalted using StageTips before analysis by MS (Rappsilber et al., 2003).

### MS analysis

A Proxeon easy-nLCII (Thermo Fisher Scientific) was directly connected to a LTQ-Orbitrap VELOS mass spectrometer (Thermo Fisher Scientific) via a nanoelectrospray ion source (Thermo Fisher Scientific). Peptides were separated by reverse phase chromatography using in-house-made C18 microcolumns (75 μm ID packed with ReproSil-Pur C18-AQ 3 μm resin, Dr. Maisch, GmbH) with a 120 min gradient from 5 to 60% acetonitrile in 0.5% acetic acid at a flow rate of 200 nL/min. Background ions were reduced using an ABIRD device (ESI Source Solutions). The LTQ-Orbitrap VELOS was operated in the data-dependent mode dynamically choosing the 10 most intense ions from the survey scan (300–1850 Th) for fragmentation using HCD. Precursor ion charge state screening was enabled and all unassigned charge states as well as singly charged species were rejected. Data were acquired using Xcalibur software.

### Data analysis

Raw data files were analyzed using MaxQuant software (version 1.1.1.27) as described (Cox and Mann, 2008). Andromeda, a probabilistic search engine incorporated into the MaxQuant framework (Cox et al., 2010), was used to search the peak lists against the mouse IPI database version 3.68. Enzyme specificity was set to trypsin and a maximum of three missed cleavages were allowed. Carbamidomethyl cysteine was set as a fixed modification, N-terminal acetylation and methionine oxidation as variable modifications. The false positive rate was set to 1% at the peptide and 1% at the protein level with a minimum required peptide length of 6 amino acids. Proteins quantified by less than two ratio counts and frequently observed contaminants were removed.

## References

[B1] Bosisio D. Polentarutti N. Sironi M. Bernasconi S. Miyake K. Webb G. R. (2002). Stimulation of toll-like receptor 4 expression in human mononuclear phagocytes by interferon-gamma: a molecular basis for priming and synergism with bacterial lipopolysaccharide. Blood 99, 3427–3431 10.1182/blood.V99.9.3427 11964313

[B2] Caminschi I. Proietto A. I. Ahmet F. Kitsoulis S. Shin Teh J. Lo J. C. (2008). The dendritic cell subtype-restricted C-type lectin Clec9A is a target for vaccine enhancement. Blood 112, 3264–3273 10.1182/blood-2008-05-155176 18669894 PMC2569177

[B3] Cox J. Mann M. (2008). MaxQuant enables high peptide identification rates, individualized p.p.b.-range mass accuracies and proteome-wide protein quantification. Nat. Biotechnol. 26, 1367–1372 10.1038/nbt.1511 19029910

[B4] Cox J. Neuhauser N. Michalski A. Scheltema R. A. Olsen J. V. Mann M. (2010). Andromeda: a peptide search engine integrated into the MaxQuant environment. J. Proteome. Res. 10, 1794–1805 10.1021/pr101065j 21254760

[B5] Ebihara S. Endo S. Ito K. Ito Y. Akiyama K. Obinata M. (2004). Immortalized dendritic cell line with efficient cross-priming ability established from transgenic mice harboring the temperature-sensitive SV40 large T-antigen gene. J. Biochem. 136, 321–328 10.1093/jb/mvh120 15598888

[B6] Edwards A. D. Diebold S. S. Slack E. M. Tomizawa H. Hemmi H. Kaisho T. (2003). Toll-like receptor expression in murine DC subsets: lack of TLR7 expression by CD8 alpha+ DC correlates with unresponsiveness to imidazoquinolines. Eur. J. Immunol. 33, 827–833 10.1002/eji.200323797 12672047

[B7] Fowlis D. J. Cui W. Johnson S. A. Balmain A. Akhurst R. J. (1996). Altered epidermal cell growth control in vivo by inducible expression of transforming growth factor beta 1 in the skin of transgenic mice. Cell Growth Differ. 7, 679–687 8732677

[B8] Fuertes Marraco S. A. Scott C. L. Bouillet P. Ives A. Masina S. Vremec D. (2011). Type I interferon drives dendritic cell apoptosis via multiple BH3-only proteins following activation by PolyIC in vivo. PLoS ONE 6, e20189 10.1371/journal.pone.0020189 21674051 PMC3107228

[B9] Hildner K. Edelson B. T. Purtha W. E. Diamond M. Matsushita H. Kohyama M. (2008). Batf3 deficiency reveals a critical role for CD8alpha+ dendritic cells in cytotoxic T cell immunity. Science 322, 1097–1100 10.1126/science.1164206 19008445 PMC2756611

[B10] Hochrein H. Shortman K. Vremec D. Scott B. Hertzog P. O’keeffe M. (2001). Differential production of IL-12, IFN-alpha, and IFN-gamma by mouse dendritic cell subsets. J. Immunol. 166, 5448–5455 11313382 10.4049/jimmunol.166.9.5448

[B11] Huysamen C. Willment J. A. Dennehy K. M. Brown G. D. (2008). CLEC9A is a novel activation C-type lectin-like receptor expressed on BDCA3+ dendritic cells and a subset of monocytes. J. Biol. Chem. 283, 16693–16701 10.1074/jbc.M709923200 18408006 PMC2562446

[B12] Inaba K. Swiggard W. J. Steinman R. M. Romani N. Schuler G. Brinster C. (2009). Isolation of dendritic cells. Curr. Protoc. Immunol. Chapter 3, Unit 3 7. 10.1002/0471142735.im0307s86 19653207

[B13] Lin M. L. Zhan Y. Proietto A. I. Prato S. Wu L. Heath W. R. (2008). Selective suicide of cross-presenting CD8+ dendritic cells by cytochrome c injection shows functional heterogeneity within this subset. Proc. Natl. Acad. Sci. U.S.A. 105, 3029–3034 10.1073/pnas.0710725105 18272486 PMC2268579

[B14] Luber C. A. Cox J. Lauterbach H. Fancke B. Selbach M. Tschopp J. (2010). Quantitative proteomics reveals subset-specific viral recognition in dendritic cells. Immunity 32, 279–289 10.1016/j.immuni.2010.01.013 20171123

[B15] Macagno A. Napolitani G. Lanzavecchia A. Sallusto F. (2007). Duration, combination and timing: the signal integration model of dendritic cell activation. Trends Immunol. 28, 227–233 10.1016/j.it.2007.03.008 17403614

[B16] Martinez del Hoyo G. Martin P. Arias C. F. Marin A. R. Ardavin C. (2002). CD8alpha+ dendritic cells originate from the CD8alpha − dendritic cell subset by a maturation process involving CD8alpha, DEC-205, and CD24 up-regulation. Blood 99, 999–1004 10.1182/blood.V99.3.999 11807005

[B17] Mazurier F. Fontanellas A. Salesse S. Taine L. Landriau S. Moreau-Gaudry F. (1999). A novel immunodeficient mouse model – RAG2 x common cytokine receptor gamma chain double mutants – requiring exogenous cytokine administration for human hematopoietic stem cell engraftment. J. Interferon Cytokine Res. 19, 533–541 10.1089/107999099313983 10386866

[B18] Merad M. Manz M. G. (2009). Dendritic cell homeostasis. Blood 113, 3418–3427 10.1182/blood-2008-12-180646 19176316 PMC2668851

[B19] Mohty M. Gaugler B. Olive D. (2003). Generation of leukemic dendritic cells from patients with acute myeloid leukemia. Methods Mol. Biol. 215, 463–471 12512320 10.1385/1-59259-345-3:463

[B20] Mortellaro A. Urbano M. Citterio S. Foti M. Granucci F. Ricciardi-Castagnoli P. (2009). Generation of murine growth factor-dependent long-term dendritic cell lines to investigate host-parasite interactions. Methods Mol. Biol. 531, 17–27 10.1007/978-1-59745-396-7_2 19347308

[B21] Naik S. H. Metcalf D. Van Nieuwenhuijze A. Wicks I. Wu L. O’keeffe M. (2006). Intrasplenic steady-state dendritic cell precursors that are distinct from monocytes. Nat. Immunol. 7, 663–671 10.1038/nrg1954 16680143

[B22] Naik S. H. Proietto A. I. Wilson N. S. Dakic A. Schnorrer P. Fuchsberger M. (2005). Cutting edge: generation of splenic CD8+ and CD8− dendritic cell equivalents in Fms-like tyrosine kinase 3 ligand bone marrow cultures. J. Immunol. 174, 6592–6597 15905497 10.4049/jimmunol.174.11.6592

[B23] Napolitani G. Rinaldi A. Bertoni F. Sallusto F. Lanzavecchia A. (2005). Selected Toll-like receptor agonist combinations synergistically trigger a T helper type 1-polarizing program in dendritic cells. Nat. Immunol. 6, 769–776 10.1038/ni1223 15995707 PMC3760217

[B24] Ong S. E. Blagoev B. Kratchmarova I. Kristensen D. B. Steen H. Pandey A. (2002). Stable isotope labeling by amino acids in cell culture, SILAC, as a simple and accurate approach to expression proteomics. Mol. Cell. Proteomics 1, 376–386 10.1074/mcp.M200025-MCP200 12118079

[B25] Poulin L. F. Salio M. Griessinger E. Anjos-Afonso F. Craciun L. Chen J. L. (2010). Characterization of human DNGR-1+BDCA3+ leukocytes as putative equivalents of mouse CD8alpha+ dendritic cells. J. Exp. Med. 207, 1261–1271 10.1084/jem.20092618 20479117 PMC2882845

[B26] Pulendran B. Tang H. Denning T. L. (2008). Division of labor, plasticity, and crosstalk between dendritic cell subsets. Curr. Opin. Immunol. 20, 61–67 10.1016/j.coi.2007.10.009 18082389 PMC2346585

[B27] Rappsilber J. Ishihama Y. Mann M. (2003). Stop and go extraction tips for matrix-assisted laser desorption/ionization, nanoelectrospray, and LC/MS sample pretreatment in proteomics. Anal. Chem. 75, 663–670 10.1021/ac026117i 12585499

[B28] Reis e Sousa C. (2004). Toll-like receptors and dendritic cells: for whom the bug tolls. Semin. Immunol. 16, 27–34 10.1016/j.smim.2003.10.004 14751761

[B29] Ruiz S. Beauvillain C. Mevelec M. N. Roingeard P. Breton P. Bout D. (2005). A novel CD4-CD8alpha+CD205+CD11b- murine spleen dendritic cell line: establishment, characterization and functional analysis in a model of vaccination to toxoplasmosis. Cell. Microbiol. 7, 1659–1671 10.1111/j.1462-5822.2005.00583.x 16207252

[B30] Salmon P. Trono D. (2007). Production and titration of lentiviral vectors. Curr. Protoc. Hum. Genet. Chapter 12, Unit 12 10. 10.1002/0471142905.hg1210s54 18428406

[B31] Sancho D. Mourao-Sa D. Joffre O. P. Schulz O. Rogers N. C. Pennington D. J. (2008). Tumor therapy in mice via antigen targeting to a novel, DC-restricted C-type lectin. J. Clin. Invest. 118, 2098–2110 10.1172/JCI34584 18497879 PMC2391066

[B32] Santegoets S. J. Van Den Eertwegh A. J. Van De Loosdrecht A. A. Scheper R. J. De Gruijl T. D. (2008). Human dendritic cell line models for DC differentiation and clinical DC vaccination studies. J. Leukoc. Biol. 84, 1364–1373 10.1189/jlb.1107750 18664532

[B33] Schulz O. Edwards A. D. Schito M. Aliberti J. Manickasingham S. Sher A. (2000). CD40 triggering of heterodimeric IL-12 p70 production by dendritic cells in vivo requires a microbial priming signal. Immunity 13, 453–462 10.1016/S1074-7613(00)00045-5 11070164

[B34] Segura J. M. Guillaume P. Mark S. Dojcinovic D. Johannsen A. Bosshard G. (2008). Increased mobility of major histocompatibility complex I-peptide complexes decreases the sensitivity of antigen recognition. J. Biol. Chem. 283, 24254–24263 10.1074/jbc.M803549200 18579518 PMC3259769

[B35] Shen Z. Reznikoff G. Dranoff G. Rock K. L. (1997). Cloned dendritic cells can present exogenous antigens on both MHC class I and class II molecules. J. Immunol. 158, 2723–2730 9058806

[B36] Shevchenko A. Wilm M. Vorm O. Mann M. (1996). Mass spectrometric sequencing of proteins silver-stained polyacrylamide gels. Anal. Chem. 68, 850–858 10.1021/ac950914h 8779443

[B37] Shortman K. Heath W. R. (2010). The CD8+ dendritic cell subset. Immunol. Rev. 234, 18–31 10.1111/j.0105-2896.2009.00870.x 20193009

[B38] Shortman K. Liu Y. J. (2002). Mouse and human dendritic cell subtypes. Nat. Rev. Immunol. 2, 151–161 10.1038/nri746 11913066

[B39] Shortman K. Naik S. H. (2007). Steady-state and inflammatory dendritic-cell development. Nat. Rev. Immunol. 7, 19–30 10.1038/nri1996 17170756

[B40] Snijders A. Kalinski P. Hilkens C. M. Kapsenberg M. L. (1998). High-level IL-12 production by human dendritic cells requires two signals. Int. Immunol. 10, 1593–1598 10.1093/intimm/10.11.1593 9846688

[B41] Steiner Q. G. Otten L. A. Hicks M. J. Kaya G. Grosjean F. Saeuberli E. (2008). In vivo transformation of mouse conventional CD8alpha+ dendritic cells leads to progressive multisystem histiocytosis. Blood 111, 2073–2082 10.1182/blood-2007-06-097576 18029555

[B42] Steinman R. M. (2008). Dendritic cells in vivo: a key target for a new vaccine science. Immunity 29, 319–324 10.1016/j.immuni.2008.08.001 18799140

[B43] van Helden S. F. Van Leeuwen F. N. Figdor C. G. (2008). Human and murine model cell lines for dendritic cell biology evaluated. Immunol. Lett. 117, 191–197 10.1016/j.imlet.2008.02.003 18384885

[B44] Villadangos J. A. Schnorrer P. (2007). Intrinsic and cooperative antigen-presenting functions of dendritic-cell subsets in vivo. Nat. Rev. Immunol. 7, 543–555 10.1038/nri2103 17589544

[B45] Villadangos J. A. Shortman K. (2010). Found in translation: the human equivalent of mouse CD8+ dendritic cells. J. Exp. Med. 207, 1131–1134 10.1084/jem.20100985 20513744 PMC2882838

[B46] Vremec D. O’keeffe M. Wilson A. Ferrero I. Koch U. Radtke F. (2011). Factors determining the spontaneous activation of splenic dendritic cells in culture. Innate Immun. 17, 338–352 10.1177/1753425910371396 20501515

[B47] Wilson N. S. Behrens G. M. Lundie R. J. Smith C. M. Waithman J. Young L. (2006). Systemic activation of dendritic cells by Toll-like receptor ligands or malaria infection impairs cross-presentation and antiviral immunity. Nat. Immunol. 7, 165–172 10.1038/ni1300 16415871

[B48] Winzler C. Rovere P. Rescigno M. Granucci F. Penna G. Adorini L. (1997). Maturation stages of mouse dendritic cells in growth factor-dependent long-term cultures. J. Exp. Med. 185, 317–328 10.1084/jem.185.2.317 9016880 PMC2196118

